# Transport, functions, and interaction of calcium and manganese in plant organellar compartments

**DOI:** 10.1093/plphys/kiab122

**Published:** 2021-03-12

**Authors:** Jie He, Nico Rössner, Minh T T Hoang, Santiago Alejandro, Edgar Peiter

**Affiliations:** Faculty of Natural Sciences III, Plant Nutrition Laboratory, Institute of Agricultural and Nutritional Sciences, Martin Luther University Halle-Wittenberg, D-06099 Halle (Saale), Germany

## Abstract

Calcium (Ca^2+^) and manganese (Mn^2+^) are essential elements for plants and have similar ionic radii and binding coordination. They are assigned specific functions within organelles, but share many transport mechanisms to cross organellar membranes. Despite their points of interaction, those elements are usually investigated and reviewed separately. This review takes them out of this isolation. It highlights our current mechanistic understanding and points to open questions of their functions, their transport, and their interplay in the endoplasmic reticulum (ER), vesicular compartments (Golgi apparatus, *trans*-Golgi network, pre-vacuolar compartment), vacuoles, chloroplasts, mitochondria, and peroxisomes. Complex processes demanding these cations, such as Mn^2+^-dependent glycosylation or systemic Ca^2+^ signaling, are covered in some detail if they have not been reviewed recently or if recent findings add to current models. The function of Ca^2+^ as signaling agent released from organelles into the cytosol and within the organelles themselves is a recurrent theme of this review, again keeping the interference by Mn^2+^ in mind. The involvement of organellar channels [e.g. glutamate receptor-likes (GLR), cyclic nucleotide-gated channels (CNGC), mitochondrial conductivity units (MCU), and two-pore channel1 (TPC1)], transporters (e.g. natural resistance-associated macrophage proteins (NRAMP), Ca^2+^ exchangers (CAX), metal tolerance proteins (MTP), and bivalent cation transporters (BICAT)], and pumps [autoinhibited Ca^2+^-ATPases (ACA) and ER Ca^2+^-ATPases (ECA)] in the import and export of organellar Ca^2+^ and Mn^2+^ is scrutinized, whereby current controversial issues are pointed out. Mechanisms in animals and yeast are taken into account where they may provide a blueprint for processes in plants, in particular, with respect to tunable molecular mechanisms of Ca^2+^ versus Mn^2+^ selectivity.

## Teaser

Calcium and manganese play essential roles in organellar compartments, yet they share many transport mechanisms, implying synergistic, and antagonistic interactions of both cations.

## Introduction

Compartmentation enables the eukaryotic cell to simultaneously carry out processes with different physicochemical requirements, such as pH, redox potential, or ion concentrations. In consequence, mineral elements fulfill specific functions in different compartments. Cellular compartments may also serve as stores for essential elements and places to safely sequester toxic compounds, with the plant vacuole representing the prime example. In any case, transport proteins are required to load and unload the compartments and thus maintain this elemental homeostasis. This review covers our current understanding of the compartmentation of two cations, calcium (Ca^2+^) and manganese (Mn^2+^), which are usually investigated and reviewed in isolation of each other. It aims to show that a more integrative view of both elements is overdue, since classical, as well as more recent work, points to an interaction of those two cations, owing to their similar coordination geometry and ionic radii. For instance, induction of Ca^2+^ deficiency symptoms and diminished Ca^2+^ translocation by excess Mn^2+^ in bean (*Phaseolus vulgaris*) plants has already been observed in the 1970s (Horst and Marschner, 1978). More recently, an interference of Mn^2+^ with Ca^2+^ translocation was confirmed for Arabidopsis and other species (Blamey et al., 2015; Lesková et al., 2017).

In plants, two primary functions for Ca^2+^ are most evident (Peiter, 2014). The bivalent cation provides structural stability by bridging carboxyl groups of galacturonans in pectin and by binding to phospholipids at cell membranes. Second, Ca^2+^ has been established as one of the universal second messengers, involved in responses to nearly every environmental cue and in a large number of developmental processes. Thereby, temporally and spatially defined elevations of free Ca^2+^, triggered by the perception of a stimulus, are sensed and “decoded” by an array of Ca^2+^-binding proteins, that either bind to or modify downstream proteins, for example, by phosphorylation. Plant Ca^2+^ signaling has been the subject of numerous recent reviews that highlight different aspects (Dodd et al., 2010; Costa et al., 2018; Kudla et al., 2018; Thor, 2019; Tian et al., 2020; Pirayesh et al., 2021). Here, we concentrate on the roles of Ca^2+^ in an organellar context, and in particular on its interaction with Mn^2+^.

An interference of Mn^2+^ in Ca^2+^ signaling is highly likely on the levels of both generation and perception of Ca^2+^ signals. Transport proteins of many families discriminate poorly between the two cations. Where known, this is highlighted for individual proteins throughout this review, and potential mechanisms of selectivity are addressed. However, for many transport processes, this interaction has not been studied yet. In animals, Ca^2+^ channels of the Ca^2+^ release-activated Ca^2+^ channel (CRAC)-, IP_3_ receptor (IP_3_R)-, and voltage-gated Ca^2+^ channel (VGCC)-type also conduct Mn^2+^ (Kamer et al., 2018). Mn^2+^ permeability is largely uncharted for Ca^2+^ channels in plants, albeit external Mn^2+^ affects Ca^2+^ influx into the cytosol (McAinsh et al., 1995; Wymer et al., 1997), and thus potentially the generation of Ca^2+^ signals. Regarding Ca^2+^ signal decoding, Mn^2+^ binds with high affinity to Ca^2+^-sensing proteins, for example, of the Calcineurin B-like (CBL) family (Sánchez-Barrena et al., 2005); the physiological effect of this competition again is unknown. Its binding to calmodulin locks the protein in a closed conformation and may, therefore, negatively regulate Ca^2+^ signaling (Senguen and Grabarek, 2012).

Our current view of Mn^2+^ in plants has been the subject of a number of comprehensive reviews (Pittman, 2005; Socha and Guerinot, 2014; Shao et al., 2017; Andresen et al., 2018; Alejandro et al., 2020). The role of Mn^2+^ considered as most important in plants is that as a redox-active constituent of the oxygen (O_2_)-evolving complex (OEC) in photosystem II (PSII), where Mn^2+^ and Ca^2+^ form a Mn_4_CaO cluster to catalyze the splitting of water (H_2_O). The chloroplast that harbors this machinery, hence, needs to accumulate large amounts of Mn^2+^, in parallel with dynamically modulating free Ca^2+^ as regulatory element, as discussed below. Further roles of Mn^2+^ are distributed amongst different compartments, such as reactive O_2_ scavenging in the mitochondria by Mn^2+^-Superoxide Dismutase (SOD), phytohormone deconjugation in the endoplasmic reticulum (ER), and glycosylation in the Golgi apparatus (Andresen et al., 2018; Alejandro et al., 2020). In yeast lacking the Golgi-localized Ca^2+^/Mn^2+^ transporter Gcr1-dependent translation factor1 (GDT1), high-Ca^2+^ media cause glycosylation defects which could be rescued by the addition of Mn^2+^ (Dulary et al., 2018), again indicating a competition of Mn^2+^ and Ca^2+^. In addition to activating enzymes, the idea of a regulatory function of Mn^2+^ in metabolism has recently been put forward (Bloom, 2019). Intriguingly, an immunity signaling mechanism based on organellar Mn^2+^ release operates in humans (Wang et al., 2018).

**Figure 1:**
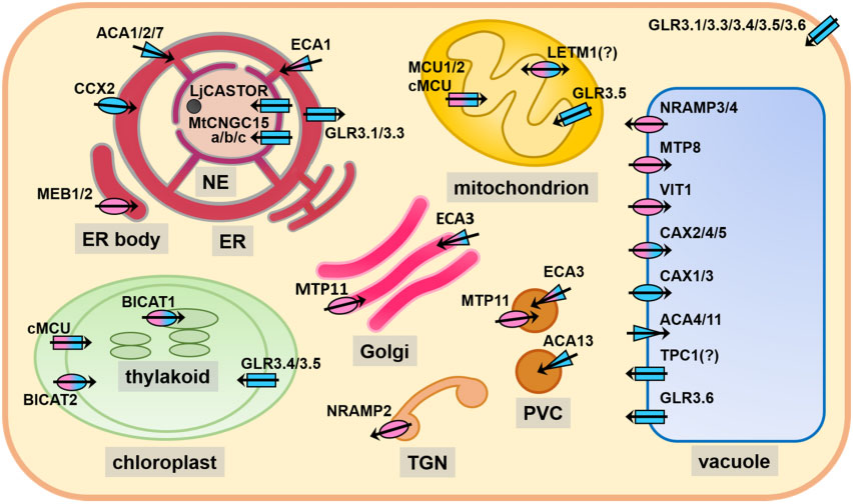
Transport proteins for Ca^2+^ and Mn^2+^ discussed in this review. Pumps (triangles), transporters (ellipses), and channels (rectangles) that were experimentally shown to permeate Ca^2+^ (blue), Mn^2+^ (magenta), or both (blue/magenta) are displayed in a hypothetical plant cell containing the organelles discussed in this review. Only Arabidopsis proteins are shown, except for LjCASTOR and MtCNGC15s, for which a function of the Arabidopsis homologs as Ca^2+^ channels has not been examined yet. Characterized orthologs of Arabidopsis proteins described in the text are listed in Table 1. Note that the absence of an experimentally confirmed substrate, for example, Mn^2+^ in the case of Ca^2+^ channels (GLR, CNGC, and TPC1) does not exclude its permeation. Conductance of Mn^2+^ by (c)MCUs is inferred from their mammalian homologs. The permeation of Ca^2+^ by NRAMPs can be excluded on a structural basis, as discussed in the text. ER, endoplasmic reticulum; NE, nuclear envelope; PVC, pre-vacuolar compartment; TGN, trans-Golgi network.

Cellular functions and interactions of Ca^2+^ and Mn^2+^ in organelles depend on their loading and unloading by an array of transport proteins from different families, which are displayed in Figure 1 and listed in Table 1. An up-to-date overview of those permeating Ca^2+^ is provided by the review of Demidchik et al. (2018); Ca^2+^ transport in the model eukaryote yeast is covered in Lange and Peiter (2020). Proteins transporting Mn^2+^ have recently been treated by Alejandro et al. (2020) and fungal mechanisms by Reddi et al. (2009). In plant organelles, primary active transport of Ca^2+^ is mediated by P_2A_- and P_2B_-type ATPases, called ER Ca^2+^-ATPases (ECAs) and Autoinhibited Ca^2+^-ATPases (ACAs), respectively. The former also transport Mn^2+^, and the latter are feedback-regulated by a Ca^2+^/calmodulin-binding autoinhibitory domain. Those pumps have recently been reviewed by Bossi et al. (2020). A large number of secondary active transporters, belonging to different families, contribute to organellar Ca^2+^ and Mn^2+^ homeostasis. The Ca^2+^ exchanger (CAX) family, reviewed by Pittman and Hirschi (2016), contains both Ca^2+^-specific transporters and those transporting Ca^2+^ and Mn^2+^; both are energized by H^+^ antiport (Waight et al., 2013). The bivalent cation transporter (BICAT) family, with its chloroplast-localized members BICAT1 and BICAT2, has been identified only recently as a family of Ca^2+^/Mn^2+^ transport proteins with a yet unresolved transport mechanism (Thines et al., 2020). The inconsistent nomenclature of these transporters is explained in the chloroplast section of this review. The founding member of this family is GDT1 in yeast (Demaegd et al., 2014). Ca^2+^ and Mn^2+^ transport have been shown directly for GDT1 (Colinet et al., 2016; Thines et al., 2018), and also for the human homolog Transmembrane protein165 (TMEM165; Stribny et al., 2020). As the mutation of these transporters causes both Ca^2+^- and Mn^2+^-related phenotypes in yeast and in plants (Thines et al., 2020), they are an ideal case to study Ca^2+^/Mn^2+^ interactions, as discussed later.

**Table 1:** Organellar transport proteins for Ca^2+^ and Mn^2+^ discussed in this review. MtCNGC15a,b,c^a^

Protein	Type	Substrates	References
**ER, NE**			
AtECA1	P_2A_ ATPase	Ca^2+^, Mn^2+^	Liang et al., 1997; Wu et al., 2002; Shkolnik et al., 2018
MtMCA8	P_2A_ ATPase	Ca^2+^, Mn^2+^ (?)^e^	Capoen et al., 2011
AtACA1, AtACA4, AtACA7	P_2B_ ATPase	Ca^2+^	Harper et al., 1998; Rahmati Ishka et al., 2021
AtCCX2	Transporter	Ca^2+^ (?)	Corso et al., 2018
AtMEB1, AtMEB2	Transporter	Mn^2+^, Fe^2+^	Yamada et al., 2013
LjCASTOR	Channel	K^+^, Ca^2+^	Charpentier et al., 2008; Kim et al., 2019
MtCNGC15^a^^b^^c^	Channel	Ca^2+^	Charpentier et al., 2016
AtCNGC15	Channel	Ca^2+^ (?)	Leitao et al., 2019
AtGLR3.1, AtGLR3.3^a^	Channel	Ca^2+^	Nguyen et al., 2018
**Golgi Apparatus, PVC, TGN**			
AtECA3	P_2A_ ATPase	Ca^2+^, Mn^2+^	Li et al., 2008; Mills et al., 2008
AtMTP11	Transporter	Mn^2+^	Delhaize et al., 2007; Peiter et al., 2007a
OsMTP11	Transporter	Mn^2+^	Farthing et al., 2017; Ma et al., 2018
AtNRAMP2	Transporter	Mn^2+^	Alejandro et al., 2017; Gao et al., 2018
**Vacuole**			
AtACA4, AtACA11	P_2B_ ATPase	Ca^2+^	Geisler et al., 2000; Lee et al., 2007; Hilleary et al., 2020
AtCAX1, AtCAX3	Transporter	Ca^2+^	Hirschi et al., 1996; Conn et al., 2011; Punshon et al., 2012
AtCAX2	Transporter	Ca^2+^, Mn^2+^, Cd^2+^	Hirschi et al., 2000; Schaaf et al., 2002; Pittman et al., 2004
AtCAX4	Transporter	Ca^2+^, Mn^2+^, Cd^2+^	Cheng et al., 2002; Mei et al., 2009
AtCAX5	Transporter	Ca^2+^, Mn^2+^	Edmond et al., 2009
OsCAX1a	Transporter	Ca^2+^, Mn^2+^	Kamiya et al., 2005; 2006
VCAX1	Transporter	Ca^2+^	Ueoka-Nakanishi et al., 2000
VvCAX3	Transporter	Ca^2+^, Mn^2+^	Martins et al., 2017
AtMTP8	Transporter	Mn^2+^, Fe^2+^	Eroglu et al., 2016, 2017; Chu et al., 2017
OsMTP8.1, OsMTP8.2	Transporter	Mn^2+^	Chen et al., 2013; Takemoto et al., 2017
ShMTP8	Transporter	Mn^2+^	Delhaize et al., 2003
AtNRAMP3, AtNRAMP4	Transporter	Mn^2+^, Fe^2+^, Cd^2+^	Thomine et al., 2003; Lanquar et al., 2010
TcNRAMP3, TcNRAMP4	Transporter	Mn^2+^, Fe^2+^, Cd^2+^	Oomen et al., 2009
AtVIT1	Transporter	Mn^2+^, Fe^2+^	Kim et al., 2006
OsVIT1, OsVIT2	Transporter	Mn^2+^, Fe^2+^, Zn^2+^	Zhang et al., 2012b
TaVIT1	Transporter	Mn^2+^, Fe^2+^	Connorton et al., 2017
AtZIP1	Transporter	Mn^2+^, Zn^2+^	Milner et al., 2013
AtGLR3.6^a^	Channel	Ca^2+^	Nguyen et al., 2018
AtTPC1	Channel	Ca^2+^, K^+^, Mg^2+^	Hedrich et al., 1986; Peiter et al., 2005
**Chloroplast**			
AtBICAT1^b^	Transporter	Ca^2+^, Mn^2+^	Schneider et al., 2016; Wang et al., 2016; Frank et al., 2019
AtBICAT2^c^	Transporter	Ca^2+^, Mn^2+^	Eisenhut et al., 2018; Zhang et al., 2018; Frank et al., 2019
AtGLR3.4^a^	Channel	Ca^2+^ (?)	Teardo et al., 2011
AtGLR3.5^a^	Channel	Ca^2+^ (?)	Teardo et al., 2015
AtcMCU^d^	Channel	Ca^2+^, Mn^2+^ (?)	Teardo et al., 2019
**Mitochondrion**			
AtGLR3.5^a^	Channel	Ca^2+^ (?)	Teardo et al., 2015
AtMCU1^d^	Channel	Ca^2+^, Mn^2+^ (?)	Teardo et al., 2017; Selles et al., 2018
AtMCU2^d^	Channel	Ca^2+^, Mn^2+^ (?)	Selles et al., 2018
AtcMCU^d^	Channel	Ca^2+^, Mn^2+^ (?)	Teardo et al., 2019

Note that the absence of an experimentally confirmed substrate, for example, Mn^2+^ in the case of Ca^2+^ channels, does not exclude its permeation.

^a^ In other studies, localization and function of AtGLR3.1, 3.3, 3.4, 3.5, and 3.6 in the plasma membrane have been demonstrated. For details, see text.

^b^ BICAT1 has also been named PAM71 and CCHA1.

^c^ BICAT2 has also been named PAM71-HL and CMT1.

^d^ Conductance of Mn^2+^ by (c)MCUs is inferred from their mammalian homologs.

^e^ (?), Substrates inferred from homologous proteins and not experimentally determined.

Members of the cation diffusion facilitator (CDF) family, called metal tolerance proteins (MTPs) in plants, mediate the H^+^-driven active export of metals, including Mn^2+^, out of the cytosol into organelles (Montanini et al., 2007; Gustin et al., 2011; Ricachenevsky et al., 2013). Transport of Mn^2+^, besides Fe^2+^, into the vacuolar organelle is also mediated by transporters of the vacuolar iron transporter (VIT) family (Kim et al., 2006). Natural resistance-associated macrophage proteins (NRAMP), in turn, import metals, including Mn^2+^, from the apoplast or from organelles into the cytosol (Nevo and Nelson, 2006). The ZRT-, IRT-like protein (ZIP) family also harbors proteins that transport Mn^2+^ across organellar membranes (Milner et al., 2013). For many Mn^2+^ transporters, Ca^2+^ transport has not been examined yet, and cannot be excluded. An exception may be NRAMP proteins, for which a coordination of Ca^2+^ was excluded on a structural basis (Ehrnstorfer et al., 2014). Within the metal-binding site, the sulfur of a conserved methionine selects against transport of Ca^2+^, while allowing the permeation of transition metals, including Mn^2+^ (Bozzi et al., 2016). All plant NRAMPs except NRAMP5 contains this selectivity motif.

The release of Ca^2+^, and potentially also Mn^2+^, from organelles along their electrochemical gradient is mediated by channel proteins. Although pharmacological analyses have frequently pointed to a contribution of organellar Ca^2+^ release to the generation of Ca^2+^ signals, the respective Ca^2+^ stores and the identity of the channels have remained mostly undefined (e.g. Thor and Peiter, 2014). Beginning with the identification of the vacuolar two-pore channel1 (TPC1; Peiter et al., 2005), a limited number of Ca^2+^-permeable channels in organellar membranes, which belong to the families of cyclic nucleotide-gated channels (CNGCs), glutamate receptor-likes (GLRs), mitochondrial conductivity units (MCUs), and possibly annexins have been uncovered (Demidchik et al., 2018). This review will indicate the relevance of those families in a physiological context.

## Ca^2+^ and Mn^2+^ in the ER and the nuclear envelope

### Functions of Ca^2+^

Within the ER of eukaryotic cells, the Ca^2+^-binding protein calreticulin is an important molecular chaperone with diverse functions (Jia et al., 2009). Unlike its animal counterpart, plant calreticulin is glycosylated. As a molecular chaperone, it mediates folding of glycoproteins and determines growth, development, and stress responses. It has been shown in animals that calreticulin modulates Ca^2+^ homeostasis due to its high capacity for Ca^2+^-binding. In animal cells, the ER indeed represents the main intracellular Ca^2+^ store for the generation of cytosolic Ca^2+^ signals. Inverse changes of [Ca^2+^]_cyt_ and [Ca^2+^]_ER_ have been directly demonstrated by simultaneous imaging of reporters targeted to both compartments (Palmer et al., 2004). Due to its reticulate structure, the ER is predestined to mediate localized Ca^2+^ fluxes, which are a prerequisite for spatio-temporally specific [Ca^2+^]_cyt_ signals. Polarization of Ca^2+^ fluxes across ER membranes allows some animal cell types to employ the ER as intracellular “Ca^2+^ tunnel” (Petersen et al., 2017), a mechanism that has not been demonstrated in plants for Ca^2+^ yet, but, intriguingly, for Zn^2+^ (Sinclair et al., 2018).

In plants, due to the overwhelming size of the vacuole, the role of the ER in Ca^2+^ signaling has been taken little into consideration in the past. However, this picture is currently changing. Using an ER-targeted Ca^2+^ reporter, distinct changes in [Ca^2+^]_ER_ upon stimulation have been revealed (Bonza et al., 2013). Unlike in animals, [Ca^2+^]_ER_ in plants increased upon [Ca^2+^]_cyt_ transients, which implies a role of the ER as Ca^2+^ buffer rather than Ca^2+^ source (Bonza et al., 2013). It has recently become apparent that [Ca^2+^]_ER_ homeostasis determines NaCl-induced [Ca^2+^]_cyt_ signals and plant sensitivity to salt stress (Corso et al., 2018). Furthermore, the phloem ER has been identified as Ca^2+^ store mediating a long-distance [Ca^2+^]_cyt_ wave in response to H_2_O potential gradients, hence determining the root growth toward H_2_O (Shkolnik et al., 2018). Specifically in some leguminous plants, Ca^2+^ release from the phloem ER plays a further role in the swelling of forisomes, proteins that expand upon Ca^2+^ binding and in turn block sieve tubes in response to disturbances (Furch et al., 2009).

The ER is continuous with the nuclear envelope (NE), which engulfs the nucleus. The steady-state [Ca^2+^] in the nucleus is similar to that in the cytosol and increases transiently or repetitively upon perception of environmental and developmental stimuli (Mazars et al., 2009), including pathogen-associated molecular patterns (PAMPs; Lecourieux et al., 2005) or microbial-derived symbiotic molecules, such as Nod factors that initiate symbiosis with rhizobial bacteria (Sieberer et al., 2009). The topic of nuclear Ca^2+^ signaling has recently been covered in an excellent review (Charpentier, 2018). Nuclear Ca^2+^ transients often follow cytosolic ones, but nuclei have also been demonstrated to be autonomous in the generation of Ca^2+^ signals, what requires Ca^2+^ release from the NE. Nuclear Ca^2+^ signals, whose kinetics differ with different stimuli, can be decoded by Ca^2+^-regulated protein kinases, such as CCaMK in the initiation of symbioses (Singh and Parniske, 2012). Besides their central position in symbiotic signaling, nuclear Ca^2+^ signatures also regulate root development (Leitao et al., 2019). As discussed below, Ca^2+^-permeable channels and transporters that generate those Ca^2+^ signals have been identified.

### Functions of Mn^2+^

In the ER, Mn^2+^ is required for phytohormone balance. Auxin amidohydrolases are Mn^2+^-dependent enzymes that participate in the activation of auxin by hydrolysis of IAA-Ala, -Leu, and -Phe into biologically active IAA (LeClere et al., 2002). In Arabidopsis, the auxin amidohydrolase family consists of seven members, of which ILR1, ILL1, ILL2, and IAR3 are located in the ER (Sanchez Carranza et al., 2016). *ilr1iar3ill2* mutant seeds and seedlings have reduced levels of free IAA and accumulate more IAA conjugates, indicating that amidohydrolases contribute free IAA to the auxin pool during germination (Rampey et al., 2004). Interestingly, IAR3 is also involved in the hydrolysis of jasmonic acid (JA)-Ile, which is the bioactive form of the phytohormone jasmonate (Widemann et al., 2013). Thus, the ER might also represent a site of signaling crosstalk between auxin and jasmonate pathways dependent on Mn^2+^.

Another role of Mn^2+^ in the ER lies in purine degradation (i.e. the ureide pathway), which can be divided into two phases. In peroxisomes, the pyrimidine ring of uric acid is cleaved to produce (S)-allantoin. In the second phase, (S)-allantoin is converted into glyoxylate in a stepwise manner by four different enzymes located in the ER (Werner and Witte, 2011). Three of these enzymes, that is, allantoate amidohydrolase (Werner et al., 2008), (S)-ureidoglycine aminohydrolase (Shin et al., 2012), and (S)-ureidoglycolate amidohydrolase (Shin et al., 2014), require Mn^2+^ as cofactor. Thus, the last part of uric acid degradation to ammonia and glyoxylate is a Mn^2+^-dependent pathway in plants.

### Import of Ca^2+^ and Mn^2+^

Active import of Ca^2+^ into the plant ER is likely mediated by P_2A_- and P_2B_-type ATPases, of which ECA1 (Liang et al., 1997) and ACA1, 2, and 7 (Harper et al., 1998; Rahmati Ishka et al., 2021) have been localized to this compartment.

ACA1, 2, and 7 are functionally redundant (Rahmati Ishka et al., 2021). Loss of the three genes caused a defect in pollen transmission and aberrances in immunity, such as salicylic acid-dependent lesions in leaves, a phenotype also found in plants devoid of vacuolar ACAs (see below). As both, fertilization and immunity are regulated by [Ca^2+^]_cyt_, the phenotypes indicate a role of the pumps in Ca^2+^ sequestration by the ER. This notion is supported by markedly increased [Ca^2+^]_cyt_ responses in the mutants to blue light and to the PAMP flg22.

Knockout mutants for *ECA1* are sensitive to low-Ca^2+^ media (Wu et al., 2002). A role of this pump in cytosolic Ca^2+^ signaling has recently been shown in Arabidopsis, where its activity shapes systemic Ca^2+^ signals triggered by a H_2_O potential gradient (Shkolnik et al., 2018). ECA1 physically interacts with MIZ1 and is inhibited by this protein (Shkolnik et al., 2018). A homolog of ECA1 has been described in rice, where it is upregulated by drought (Huda et al., 2013). This may point to a similar role in H_2_O tracking as it has been shown in Arabidopsis. In *Medicago truncatula*, an ER- and NE-localized ECA is essential for the generation of nuclear Ca^2+^ oscillations upon Nod factor perception, and it determines symbiotic establishment (Capoen et al., 2011). Sequestration of Ca^2+^ by ATPases is thus crucial in diverse Ca^2+^ signaling scenarios.

ECA1 is the only transport protein described so far that moves Mn^2+^ into the plant ER (Liang et al., 1997; Johnson et al., 2009). ECA2 and ECA4 contain an ER retention motif, but have not been functionally characterized yet (Bossi et al., 2020). Arabidopsis *eca1* mutants show growth inhibition by high Mn^2+^, indicating that ECA1 is important for tolerance to toxic levels of Mn (Wu et al., 2002). The absence of a phenotype under standard growth conditions suggests the existence of other mechanisms responsible for Mn^2+^ transport into the ER, with ECA2 and ECA4 being suitable candidates. Since ECA pumps are a major factor in Ca^2+^ and Mn^2+^ homeostasis, yet unknown mechanisms must exist to discriminate between both ions.

Apart from P-type ATPases, an ER-localized transporter of the Ca^2+^/cation exchanger family, CCX2, contributes to Ca^2+^ homeostasis in ER and cytosol, thereby modulating abiotic stress responses (Corso et al., 2018). Free Ca^2+^ in the ER is increased and concentrations in cytosol are decreased upon NaCl treatment if CCX2 is absent, which implies that CCX2 either transports Ca^2+^ itself or regulates a Ca^2+^ transport protein.

Specific ER subcompartments, named ER bodies, contain two Mn^2+^/Fe^2+^ transporters, membrane protein of ER body 1 (MEB1) and MEB2, that are distantly related to the vacuolar transporter VIT1 (Yamada et al., 2013). ER bodies have been found only in Brassicales (Matsushima et al., 2003), where they are constitutively present in roots and induced by wounding or jasmonate treatment in epidermal cells of mature leaves. It has been proposed that this organellar structure functions in metal sequestration and in the defense against herbivores by accumulating EE-type myrosinases (Yamada et al., 2011). Since Mn^2+^ may stimulate myrosinase activity (Ohtsuru and Kawatani, 1979), the loading of Mn^2+^ into ER bodies is likely required for their function.

### Release of Ca^2+^ and Mn^2+^

Our molecular understanding of Ca^2+^ release from the ER only partially explains biochemical findings. In animals, it is facilitated by two classes of ligand-gated channels: Ryanodine receptors (RyRs) activated by cyclic adenosine diphosphate ribose (cADPR), and IP_3_Rs. Genomes of plants do not encode these proteins. Yet, cADPR activates Ca^2+^ release from ER membrane vesicles (Navazio et al., 2001), and this molecule has been assigned a role in the circadian clock (Dodd et al., 2007), in abscisic acid signaling (Sánchez et al., 2004), and in NO-dependent responses (Abdul-Awal et al., 2016), all involving an increase of [Ca^2+^]_cyt_. Further complexity is added by a likely interference of cADPR synthesis and poly-ADP(ribosyl)ation due to their common precursor, NAD^+^ (Rissel and Peiter, 2019). The source of cADPR in plants has been completely obscure until recently, when the biosynthesis of a cADPR variant, v-cADPR, by the TIR domain of nucleotide-binding leucine-rich repeat (NLR) immune receptors was shown (Wan et al., 2019). NLR-mediated synthesis of v-cADPR promotes cell death. It is, however, still unknown if the enzymatic activity of the TIR domain is also responsible for cADPR synthesis in the circumstances mentioned above.

Besides cADPR, the NAD^+^-based nucleotide NAADP was shown to release Ca^2+^ from plant ER-derived vesicles (Navazio et al., 2000). In animals, NAADP activates TPCs resident in endolysosomal membranes (Calcraft et al., 2009). However, as discussed below, plant TPC proteins reside in the vacuolar membrane, and they are not activated by NAADP. In addition to ligand-gated Ca^2+^ conductances, voltage-gated Ca^2+^ channels have been detected by lipid bilayer electrophysiology in reconstituted plant ER membranes (Klüsener et al., 1995; Klüsener and Weiler, 1999). At present, the molecular mechanisms and physiological relevance of all those Ca^2+^ release pathways are unclear. However, the early biochemical and electrophysiological observations might be associated with channel proteins that have been localized to the ER.

Two channel proteins of the GLR family, GLR3.1 and GLR3.3, have recently been assigned a localization to ER-like structures of xylem contact cells and phloem sieve elements, respectively (Nguyen et al., 2018). In these tissues, those channels are essential for the propagation of electropotential waves and systemic Ca^2+^ signals triggered by wounding and herbivory (Farmer et al., 2020). For GLR3.3, Ca^2+^ permeability has been shown by expression in Cos-7 cells (Wudick et al., 2018) and HEK293T cells (Shao et al., 2020). The apparent localization of the channels to endomembranes cannot explain the defects of mutants for those genes, namely their inability to elicit systemic plasma membrane potential changes and [Ca^2+^]_cyt_ responses to apoplastic glutamate (Mousavi et al., 2013; Toyota et al., 2018). Furthermore, GLR3.1 functions, together with GLR3.5, as L-Met-activated Ca^2+^ channel in the plasma membrane of stomatal guard cells (Kong et al., 2016). Also, GLR3.3 has been described before to mediate Ca^2+^ entry into root cells, plasma membrane depolarization, and macroscopic currents upon stimulation with various amino acids, glutathione, or 1-aminocyclopropane-1-carboxylic acid (Qi et al., 2006; Mou et al., 2020). Accordingly, the ligand-binding domain of GLR3.3, the structure of which has recently been solved, binds glutamate and other amino acids in the micromolar range (Alfieri et al., 2020). GLR3.3 also localizes to the plasma membrane in pollen sperm cells, where its exit from the ER is regulated by CORNICHON proteins (Wudick et al., 2018). This mechanism might also control its localization in the vascular system. There, besides the reported ER localization, a low number of channels may be present in the plasma membrane that mediate the observed chemo-electrical responses (Grenzi et al., 2020). It is unclear at present if and under which circumstances GLR3.1 and 3.3 mediate Ca^2+^ release from the ER.

The generation of the above-mentioned oscillatory nuclear Ca^2+^ signals that initiate symbiotic interactions with rhizobial bacteria or mycorrhizal fungi relies on Ca^2+^ release from the NE, which is continuous with the ER (Zipfel and Oldroyd, 2017). Three CNGC15 paralogs have been identified as Ca^2+^ channels that mediate those fluxes (Charpentier et al., 2016). These channels physically interact with and are regulated by another cation channel, DMI1 in *M. truncatula*, or CASTOR/POLLUX in *Lotus japonicus*, the absence of which completely abolishes the nuclear Ca^2+^ spiking. The localization of DMI1 preferably in the inner nuclear membrane supports its role in nuclear Ca^2+^ signal generation (Capoen et al., 2011). Recently, the generation of nuclear Ca^2+^ signals in Arabidopsis, which does not enter symbiotic interactions, was demonstrated to also involve orthologs of DMI1, and possibly CNGC15 (Leitao et al., 2019). Medicago DMI1 modulates Ca^2+^ signals upon expression in yeast (Peiter et al., 2007b), and, based on lipid bilayer analyses, DMI1 and CASTOR/POLLUX are believed to primarily conduct K^+^ and to regulate Ca^2+^ oscillations by altering the NE’s membrane potential (Charpentier et al., 2008; Venkateshwaran et al., 2012). However, this view has recently been challenged by Kim et al. (2019), who reported Ca^2+^ permeability and Ca^2+^ activation of CASTOR, which would render it a Ca^2+^-induced Ca^2+^ release (CICR) channel. A large gating ring structure of the protein contains Ca^2+^-binding sites that biphasically stimulate or inhibit channel activity (Kim et al., 2019).

The Mn^2+^ permeability of ER-localized Ca^2+^ channels has not been analyzed yet, but is likely of relevance, given the fact that ECA1 accumulates Mn^2+^ into this organelle. Specific mechanisms of Mn^2+^ release from the ER in plants are also still unknown. There is neither evidence in humans nor in yeast of transporters mediating Mn^2+^ efflux from ER, although in yeast Mn^2+^ release from the ER to the Golgi via vesicular trafficking has been proposed (Garcia-Rodriguez et al., 2015).

## Ca^2+^ and Mn^2+^ in the Golgi, TGN, and PVC

### Functions of Ca^2+^

The luminal homeostasis of ions, including Ca^2+^ and Mn^2+^, next to the pH determines Golgi functions (Kellokumpu, 2019). However, our functional understanding of Ca^2+^ in the plant’s Golgi apparatus and other vesicular compartments is still scant. In the Golgi, post-translational modifications like glycosylation often require bivalent cations as cofactors. Albeit this is mostly Mn^2+^, some enzymes, such as ER-type α-mannosidase I (MNS3), employ Ca^2+^ or Mn^2+^ for their catalytic activity (Liebminger et al., 2009). In tobacco (*Nicotiana tabacum*), Ca^2+^ serves as cofactor for a Golgi-localized calreticulin, likely required for correct protein folding similarly to ER-located calreticulin (Nardi et al., 2006).

In animal cells, a [Ca^2+^] gradient exists along the compartments of the secretory pathway that is likely of functional relevance (Suzuki et al., 2016). Whereas the basal free Ca^2+^ in the ER is around 700 µM, free Ca^2+^ in the Golgi is lower, decreasing from 150–300 µM in the *cis*-Golgi to 50–100 µM in the *trans*-Golgi (Aulestia et al., 2015). Similarly, there is a pH gradient along the compartments of the animal secretory pathway (Kellokumpu, 2019). A gradient of decreasing pH is also observed along the plant secretory pathway (Shen et al., 2013), whereby an increase from Golgi to pre-vacuolar compartment (PVC) and late PVC has been observed (Martinière et al., 2013). In contrast, this has not been assessed for Ca^2+^ yet. Intriguingly, the single available report on [Ca^2+^] in the plant Golgi showed a concentration of merely 0.7 µM (Ordenes et al., 2012). This indicates that [Ca^2+^]_Golgi_ homeostasis in plants may differ substantially from that in animals. However, determination of absolute [Ca^2+^] in the secretory pathway is challenging. Albeit precise targeting to subcompartments is pursuable by genetically encoded Ca^2+^ indicators (GECIs), a formidable challenge lies in the pH dependence of most of those fluorescence-based reporters (Suzuki et al., 2016). Because of this, the luminescent reporter aequorin is still a suitable tool to report Ca^2+^ in acid compartments due to its independence of pH (Brini, 2008).

Free Ca^2+^ in the plant Golgi is responsive to various stimuli. In response to cold shock, mechanical stimulation, or hyperosmotic stress, Ordenes et al. (2012) monitored an increase in [Ca^2+^]_Golgi_ that was delayed compared to the [Ca^2+^]_cyt_ rises. This led to the conclusion that Ca^2+^ is moved into the Golgi apparatus to restore the basal [Ca^2+^]_cyt_. Apart from that, an increase in [Ca^2+^]_Golgi_ induced by environmental stimuli may regulate vesicular trafficking, which is required to cope with stresses, for example, salt stress (Leshem et al., 2006).

Secretory proteins, molecules, and lipids traverse through the secretory pathway including the Golgi apparatus, the *trans*-Golgi network (TGN), and the PVC. In plants, vacuolar sorting receptors (VSRs) are involved in cargo transport to the vacuole (Miao et al., 2006). VSRs contain epidermal growth factor (EGF)-like repeats which play a role in Ca^2+^ interaction, and the binding of vacuolar sorting determinants (VSDs) to VSRs occurs in a Ca^2+^-dependent manner (Suen et al., 2010; Robinson and Pimpl, 2014). Consequently, Künzl et al. (2016) concluded that VSDs bind to VSRs in ER and Golgi at neutral pH and high [Ca^2+^], whereas VSRs release VSDs in the TGN or in PVCs under mildly acidic pH and low [Ca^2+^]. In addition, in animals, the sorting of secretory proteins depends on the import of Ca^2+^ into the TGN by the secretory pathway Ca^2+^-ATPase1 (SPCA1) in a sphingomyelin-dependent manner (Deng et al., 2018).

Besides the potential regulatory activity of Ca^2+^ within organelles of the secretory pathway, its release will cause local [Ca^2+^]_cyt_ elevations that may be sensed by secretory pathway-localized proteins. For instance, the auxin derivate 2,4-D induced a decrease in [Ca^2+^]_Golgi_ (Ordenes et al., 2012). Several Ca^2+^-sensing proteins associated with the secretory pathway have been identified. For instance, AGD12 and EHB1, located to the TGN, contain a cytosolic Ca^2+^-binding C2 domain (Dümmer et al., 2016). AGD12 is an activator of ARF GTPases that mediate vesicular transport and membrane docking (Nielsen et al., 2008). Mutants lacking either of those proteins are affected in gravitropism, which is regulated by auxin (Dümmer et al., 2016) and [Ca^2+^]_cyt_-dependent (Monshausen et al., 2011; Shih et al., 2015). A decrease in [Ca^2+^]_Golgi_ might thus lead to local [Ca^2+^]_cyt_ elevations around the Golgi that trigger AGD12 and EHB1. Interestingly, EHB1 has recently been described to inhibit the Fe^2+^ (and Mn^2+^) uptake transporter IRT1 in a Ca^2+^-dependent way, albeit in that study, EHB1 was localized to the plasma membrane (Khan et al., 2019).

In Arabidopsis, two calmodulin-like proteins, CML4 and CML5, with yet unknown function are localized to organelles of the secretory pathway (Ruge et al., 2016). Their CaM domain faces the cytosol and will thus sense [Ca^2+^]_cyt_ elevations around those compartments. Finally, synaptotagmins (SYTs), which contain two cytosolic C2 domains, are also predestined targets of such local [Ca^2+^]_cyt_ hotspots. In animals, SYTs regulate membrane trafficking and fusion in a Ca^2+^-dependent manner (Südhof, 2013). Out of the five SYTs in Arabidopsis, SYT2 has been localized to the Golgi apparatus and suggested to be required for unconventional secretion (Zhang et al., 2011) as well as for conventional exocytosis in pollen tubes, whereby it is additionally localized at the plasma membrane (Wang et al., 2015).

### Functions of Mn^2+^

As cofactor of glycosyltransferases (GTs), Mn^2+^ plays a pivotal role in glycosylation reactions in the Golgi apparatus (Nagashima et al., 2018). GTs are classified into three groups based on their 3D folds, GT-A, GT-B, and GT-C (Moremen and Haltiwanger, 2019). Most of the GT-A fold enzymes contain conserved DxD (Asp-[any amino acid]-Asp) motifs which interact with the phosphate group of the nuclear sugar donor through the coordination of a divalent cation, which is typically Mn^2+^ (Breton et al., 2006). Although no DxD motif exists in GT-B fold GTs, some of them require a divalent cation for their optimal activity (Breton et al., 2006). For instance, Mn^2+^ strongly enhances the catalytic activity of a fucosyltransferase by increasing its affinity to receptors, even though the divalent cation is not absolutely essential for its enzymatic activity (Palma et al., 2004).

**Figure 2:**
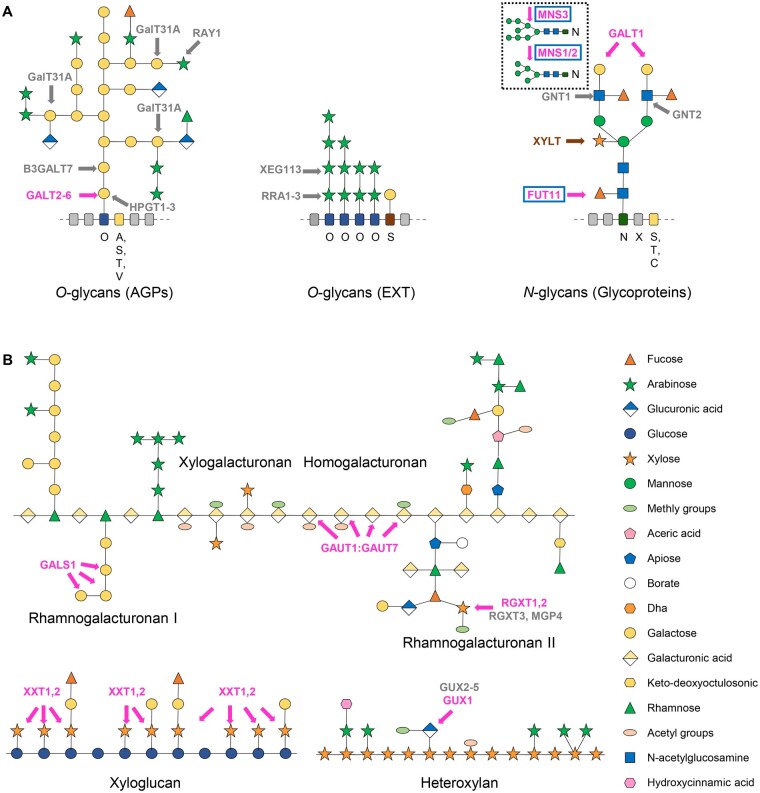
Mn^2+^ and Ca^2+^ dependence of glycosylation in the Golgi. Enzymes are either marked in magenta for those experimentally confirmed to require Mn^2+^ or in gray for those predicted to be Mn^2+^-dependent. Enzymes that are also activated by Ca^2+^ are framed in blue. An enzyme that does not absolutely depend on Mn^2+^ is marked in brown. A, Schematic representation of enzymes involved in synthesis of *O*-glycans attached to plant AGPs and EXTs, and specific complex-type *N*-glycans attached to plant glycoproteins. The glycan models presented are modified from Nguema-Ona et al. (2014) and Showalter and Basu (2016). Among those core enzymes of deglycosylation (insert) and glycosylation, MNS1, MNS2, MNS3 (Liebminger et al., 2009), and FUT11 (Both et al., 2011) are activated by Ca^2+^ or Mn^2+^. XYLT (Pagny et al., 2003), GALT1 (Strasser et al., 2007), and GALT2 to 6 (Basu et al., 2015a, 2015b) are reported to coordinate Mn^2+^ for their catalytic activity. Activity of XYLT is stimulated or inhibited by Mn^2+^ (Bencúr et al., 2005). Based on the Uniprot database (www.uniprot.org), some further Arabidopsis GTs involved in *N*- or *O*-glycosylation are predicted to contain a DxD motif or to bind Mn^2+^ by sequence similarity, that is, β-1,2-N-acetylglucosaminyltransferase (GNT1 and 2), β-1,2-arabinosyltransferase (RRA1,2,3 and XEG113), hydroxyproline-*O*-galactosyltransferase (HPGT1,2,3), β-1,3-galactosyltransferase (B3GALT7), β-1,6-galactosyltransferase (GALT31A), and β-arabinofuranosyltransferase (RAY1). B, Schematic representation of enzymes involved in the synthesis of matrix sugars of the plant cell wall. The structures of matrix sugars are modified from Burton et al. (2010). Enzymes shown to require Mn^2+^ are galactan synthase 1 (AtGALS1) that catalyzes the addition of galactose from UDP-α-D-Gal to β-1,4-galactan chains of rhamnogalacturonan I and the transfer of an arabinopyranose from UDP-β-L-Ara_*p*_ to galactan chains (Ebert et al., 2018; Laursen et al., 2018); a polygalacturonate (1,4)-α-D-galacturonosyltransferase complex (GAUT1:GAUT7) that catalyzes the transfer of galacturonic acid onto homogalacturonan (Amos et al., 2018); (1,3)-α-D-xylosyltransferases (AtRGXT1 and 2) that synthesize rhamnogalacturonan-II (RG II; Egelund et al., 2006; Petersen et al., 2009); xyloglucan xylosyltransferases (XXT1 and 2) that are involved in xyloglucan biosynthesis (Culbertson et al., 2016; 2018); and GUX1 that adds GlcA to xylan (Rennie et al., 2012). In addition, based on the Uniprot database (www.uniprot.org), further Arabidopsis GTs involved in matrix sugar biosynthesis are predicted to contain a DxD motif or to bind Mn^2+^ by sequence similarity, that is, rhamnogalacturonan α-1,3-D-xylosyltransferase (MGP4 and RGXT3) for RGII biosynthesis, and UDP-GlcA:xylan glucuronyltransferase (GUX2,3,4,5) for heteroxylan biosynthesis.

In all eukaryotes, GTs modify proteins in a complex and coordinated way (Nguema-Ona et al., 2014; Schoberer and Strasser, 2018). Thereby, *N*-linked protein glycosylation is initiated in the ER. The intermediate *N*-glycans are transmitted to the *cis*-Golgi and processed further by MNS3 to remove a mannose residue, followed by maturation while progressing through the Golgi stack from *cis*- to *trans*-face. This is accomplished by Golgi α-mannosidase I (MNS1 and MNS2), *N*-acetylglucosaminyltransferase I (GNTI), Golgi α-mannosidase II (GMII), *N*-acetylglucosaminyltransferase II (GNTII), α-1,3-fucosyltransferase (FUT11 and FUT12), β-1,2-xylosyltransferase (XYLT), β-1,3-galactosyltransferase (GALT1), and α-1,4-fucosyltransferase (FUT13; Nagashima et al., 2018). Among them, some have been shown to coordinate Mn^2+^ for their catalytic activity (Figure 2A), and mutants of them show abnormal growth phenotypes.

Extensins (EXTs) and arabinogalactan proteins (AGPs) are two major *O*-glycosylated protein components of the plant cell wall. They belong to the hydroxyproline-rich glycoprotein superfamily and account for around 10% of cell wall dry weight. AtGALT2 to 6 are Golgi-localized Hyp-*O*-galactosyltransferases, responsible for transferring galactose to hydroxyproline residues of AGPs, and require Mn^2+^ for their optimal activity (Figure 2A). Mutants show aberrant root growth, as well as seed mucilage and seed set phenotypes (Basu et al., 2015a, 2015b). Based on the Uniprot database (www.uniprot.org), some further Arabidopsis GTs involved in *O*-glycosylation are predicted to contain a DxD motif or to bind Mn^2+^ by sequence similarity (Figure 2A).

Unlike protein glycosylation, the synthesis of matrix polysaccharides (pectin and hemicellulose) takes place exclusively in the Golgi. Some of the Arabidopsis GTs involved in this process have been described to work in a Mn^2+^-dependent manner (Figure 2B). There are eight plant glycogenin-like starch initiation proteins (PGSIPs) in Arabidopsis, of which PGSIP1 to 5 were later annotated as glucuronic acid substitution of xylan1 (GUX1) to 5, respectively. Albeit initially believed to be involved in starch synthesis, *GUX1* encodes a glucuronosyltransferase mediating the addition of GlcA to xylan and requires Mn^2+^ for its catalytic activity (Rennie et al., 2012). Another member of this protein family, PGSIP6/IPUT1/MOCA1, is a showcase example for a Mn^2+^-dependent glycosylation process playing multiple crucial roles in signaling and development. Using the same sugar donor as GUX1, PGSIP6 catalyzes the transfer of a GlcA residue to glycosyl inositol phosphorylceramide (GIPC) sphingolipids and hence was renamed to inositol phosphorylceramide glucuronosyltransferase1 (IPUT1; Rennie et al., 2014). GIPCs are abundant in the plasma membrane, where they make up about a quarter of the total lipids, and also reside in tonoplast and ER membranes. Homozygous *iput1* T-DNA insertional loss-of-function mutants are lethal (Rennie et al., 2014), but expression of *IPUT1* under a pollen-specific promoter in an *iput1* knockout line allowed to generate pollen-specifically rescued homozygous *iput1* mutants (Tartaglio et al., 2017). These mutants contain fewer GIPCs and a severely altered sphingolipidome, and showed severe dwarfism, compromised pollen tube guidance, and constitutive activation of salicylic acid-mediated defense pathways, indicating important roles of GIPC sphingolipid glycosylation.

Based on its homology to GUX1, PGSIP6/IPUT1/MOCA1 is bound to be Mn^2+^-dependent, which has yet to be confirmed experimentally. In this respect, it is interesting to note that Bian et al. (2018) identified a mutant of this gene in a screen for hypersensitivity to low Mn^2+^ supply. This mutant contains a nonsynonymous point mutation which, however, is not close to the Mn^2+^-binding site of the protein, but which may cause a partial loss in enzymatic activity, leading to the Mn^2+^-dependent growth defect. It is unclear if the mutant protein requires a higher [Mn^2+^]_Golgi_ to function, or if Mn^2+^ may even have a regulatory role.

Finally, the most recent characterization of the PGSIP6/IPUT1/MOCA1 protein combines this role of Mn^2+^ with Ca^2+^ signaling. Intriguingly, a mutant called* monocation-induced [Ca^2+^]_i_ increases1* (*moca1*) that carries a four-amino acid-deletion in PGSIP6/IPUT1/MOCA1, was identified in a screen for aberrant [Ca^2+^]_cyt_ signals in response to salt (Na^+^) stress (Jiang et al., 2019). Transient [Ca^2+^]_cyt_ elevation is an early and essential response to salt stress (Dodd et al., 2010). However, the Na^+^ sensor in plants has been unknown. Due to the abolished Na^+^-triggered [Ca^2+^]_cyt_ signal in the *moca1* mutant, GIPCs synthesized by the PGSIP6/IPUT1/MOCA1 protein are believed to fulfill this role by coordinating Na^+^ to gate Ca^2+^ influx channels (Jiang et al., 2019). Taken together, this implies that Na^+^ sensing, Ca^2+^ signal generation, and hence salt tolerance, depend on a correct Mn^2+^ supply to the Golgi. Furthermore, based on the multitude of reported phenotypes in mutants of *PGSIP6/IPUT1/MOCA1*, it remains to be confirmed whether its enzymatic product indeed functions as Na^+^ sensor, or whether its role is more indirect.

The requirement of bivalent cations by GTs is likely to be more complex as generally anticipated, as the concentration of the cation is an important factor, and in some cases, cations can act in an inhibitory way. For instance, the activity of GALT5 is enhanced by Mn^2+^ but inhibited by Ca^2+^ (Basu et al., 2015b). Besides, the activity of XylT, which does not absolutely depend on metal ion cofactors, is enhanced by 1 mM Mn^2+^, while higher Mn^2+^ concentrations are inhibitory (Bencúr et al., 2005). Cation concentrations and interactions in the Golgi thus appear to modulate, or even regulate GT enzymes. Considering the specific targeting of enzymes to distinct Golgi subcompartments as well as the different cation requirements of GTs, a concerted regulation of the concentration and ratio of Ca^2+^ and Mn^2+^ in the Golgi apparatus is likely to be crucial. However, our understanding of this matter in plants is very rudimentary. In animal cells, the Ca^2+^/Mn^2+^ transporter TMEM165 is important for glycosylation, and some Ca^2+^/Mn^2+^-ATPases are also potentially involved in Ca^2+^ and Mn^2+^ supply of Golgi-localized GTs, as discussed below. In yeast, the Ca^2+^/Mn^2+^-ATPase PMR1 and the Ca^2+^/Mn^2+^ transporter GDT1 are required for protein glycosylation (Dürr et al., 1998; Colinet et al., 2016). In contrast, no cation transport protein has been associated with the maintenance of glycosylation reactions in plants.

There is evidence that the Golgi and TGN take in a central position in subcellular Mn^2+^ allocation. In yeast, SMF2, an NRAMP transporter, is assumed to be crucial for releasing Mn^2+^ from vesicular compartments (*trans*-Golgi and late endosomes) to the cytosol (Garcia-Rodriguez et al., 2015), whereby Mn^2+^ can subsequently be transferred to the mitochondria by unknown transporters and to the Golgi by PMR1. Accordingly, a Δ*smf2* mutant exhibits reduced SOD2 activity and Mn^2+^ accumulation in mitochondria, and defective glycosylation of secreted invertase (Luk and Culotta, 2001). In Arabidopsis, the TGN-localized NRAMP2 is believed to be functionally epistatic to NRAMP3 and NRAMP4, two vacuolar Mn^2+^ and Fe^2+^ transporters, involved in the redistribution of Mn^2+^ to vacuoles and chloroplasts under Mn^2+^ deficiency (Alejandro et al., 2017). Mutants for *NRAMP2* show reduced photosynthesis and Mn^2+^ concentrations in chloroplasts and vacuoles. Based on a model put forward by Krieger-Liszkay and Thomine (2018), Mn^2+^ released from TGN and vacuole to cytosol can be subsequently translocated to the chloroplast stroma by BICAT2 and further imported into the thylakoid lumen by BICAT1 to supply the H_2_O-splitting complex and maintain photosynthetic efficiency under Mn^2+^ deficiency. This implies that vesicular compartments are essential for inter-organellar Mn^2+^ distribution.

Studies in the animal field suggest a crucial role of the Golgi in Mn^2+^ storage and detoxification. By combining synchrotron X-ray fluorescence (SXRF) nanoprobe with particle-induced X-ray emission (PIXE) microanalysis and backscattering spectrometry, Carmona et al. (2010) observed an accumulation of Mn^2+^ within the Golgi of PC12 dopaminergic cells at physiological concentrations, and a further increase of the Golgi-allocated fraction when cells were exposed to 100 µM MnCl_2_, a subcytotoxic level. Upon exposure to toxic concentrations, Mn^2+^ was also detected in cytoplasm and nucleus. Based on monitoring the subcellular distribution of Mn^2+^ in living HEK293T cells by a fluorescent Mn^2+^ sensor and by nano-SXRF imaging, Das et al. (2019) obtained evidence that the Golgi, besides its function as a Mn^2+^ storage organelle under physiological conditions, has an additional trafficking role under subcytotoxic Mn^2+^ conditions. No direct measurement of compartmental Mn^2+^concentrations has been conducted in plants yet. However, a crucial role of the Golgi in Mn^2+^ detoxification has been inferred from the Mn^2+^-hypersensitive phenotype of the Golgi-localized Mn^2+^ transporter MTP11 (Peiter et al., 2007a). It would be highly interesting to develop genetically encoded Mn^2+^ indicators to monitor the subcellular Mn^2+^ distribution under different levels of Mn^2+^ supply. In analogy to GECIs, specificity to Mn^2+^ may be conferred by a Mn^2+^-specific binding domain, for example from the cyanobacterial Mn^2+^ sensor MntS (Yamaguchi et al., 2002). In combination with a more complete inventory of Golgi-localized Mn^2+^ transport proteins, this would help to elucidate poorly understood plant Golgi functions, such as different Mn^2+^ requirements of complex glycosylation processes, inter-organellar Mn^2+^ transit, Mn^2+^ storage and detoxification, and, in particular, Mn^2+^–Ca^2+^ interactions.

### Import of Ca^2+^ and Mn^2+^

In animals and fungi, the import of Ca^2+^ and Mn^2+^ into the Golgi apparatus is mediated by pumps and transporters, whose cooperative function may ensure optimal supply. The P_2A_-type ATPases SERCA2 and SPCA1 and 2 are localized to the Golgi apparatus of animal cells with specific distribution across the cisternae (Wong et al., 2013; Pizzo et al., 2011). In addition, SPCA1 is localized to the TGN, where it was shown to determine vesicular transport of secretory proteins (Deng et al., 2018). Yeast owns a sole P_2A_ pump, PMR1. Both PMR1 and SPCA1 have structural features that determine Ca^2+^ versus Mn^2+^ selectivity. SPCA1 contains an N-terminal Ca^2+^-binding EF-hand motif, which increases the relative turnover rate of Ca^2+^ (Chen et al., 2019). A similar mechanism to modulate substrate specificity is present in PMR1 (Wei et al., 1999), in addition to other factors based on sequence and structure that determine selectivity (Mandal et al., 2000, 2003). These Golgi-localized ATPases thus appear to be equipped with mechanisms to specifically modulate Mn^2+^ and Ca^2+^ transport, which may be important to provide the optimal cation concentrations for the processes described above. Apart from supplying the Golgi for glycosylation reactions, PMR1 is a Mn^2+^ tolerance factor by clearing cytosolic Mn^2+^, which is then secreted by exocytosis (Dürr et al., 1998).

Besides ATP-driven pumps, members of a recently identified transporter family, CaCA2, determine Ca^2+^ and Mn^2+^ homeostasis in the Golgi: TMEM165 in humans and GDT1 in yeast. Initially believed to be Ca^2+^/H^+^ antiporters that regulate Ca^2+^ and pH homeostasis (Demaegd et al., 2013), they were subsequently shown to transport also Mn^2+^ (Stribny et al., 2020). This renders them crucial for Golgi-localized Mn^2+^-dependent glycosylation processes (Potelle et al., 2016; Foulquier and Legrand, 2020). Interestingly, TMEM165 and GDT1 themselves are sensitive to Mn^2+^ and degraded upon elevated levels of cytosolic Mn^2+^ (Potelle et al., 2017; Dulary et al., 2018). An abolition of SPCA1-mediated Mn^2+^ loading of the Golgi thus entails the shut-down of the alternative TMEM165 pathway to supply the Golgi with Mn^2+^. This astonishing interaction might serve to protect the Golgi from Mn^2+^ overload that might interfere with Ca^2+^-dependent processes. The interaction of Ca^2+^ and Mn^2+^ transport by TMEM165 and GDT1 is not completely understood yet, and it was even suggested that the proteins may mediate a Ca^2+^/Mn^2+^ antiport (Dulary et al., 2018).

As compared to the Ca^2+^ and Mn^2+^ supply of animal and yeast Golgi, information on plants is even more rudimentary. One P_2A_-type ATPase, ECA3, is localized in the plant secretory pathway, and suggested to function as Ca^2+^/Mn^2+^ pump as shown in yeast complementation experiments. Two studies localized it to the Golgi and the PVC (Li et al., 2008; Mills et al., 2008). An involvement in Ca^2+^ and Mn^2+^ homeostasis is supported by phenotypes of *eca3* mutants, which are hypersensitive to low Ca^2+^ or high Mn^2+^ supply. The protein was thus suggested to detoxify Mn^2+^ via loading it into vesicles followed by exocytosis, and to ensure Ca^2+^-dependent processes in the Golgi (Li et al., 2008). Potential regulation of this pump by Ca^2+^ or Mn^2+^ has yet to be studied.

Similar to ECA3, MTP11, a member of the CDF family, was localized to the Golgi (Peiter et al., 2007a) and the PVC (Delhaize et al., 2007). Its capacity to transport Mn^2+^ by Mn^2+^/H^+^ antiport was shown by direct transport assays (Delhaize et al., 2007; Peiter et al., 2007a). *mtp11* knockout mutants are hypersensitive and *MTP11* overexpressors hypertolerant to Mn^2+^ toxicity. Since knockout mutants accumulated more Mn^2+^, Peiter et al. (2007a) suggested a mechanism of Mn^2+^ detoxification by MTP11-mediated Mn^2+^ loading of vesicles followed by exocytosis. As an equivalent mechanism has been suggested for ECA3, it ought to be analyzed if both proteins operate in the same pathway or even interact. Interestingly, *mtp11* mutants were hypertolerant to low Mn^2+^ supply, indicating that the supply of critical Mn^2+^-dependent processes was improved.

Orthologs of Arabidopsis MTP11 from poplar (PtdMTP11.1/2) and rice (OsMTP11) also complemented a Mn^2+^-sensitive yeast strain deleted in *PMR1*, and OsMTP11 rescued the Mn^2+^ sensitivity of an Arabidopsis *mtp11* mutant, suggesting the functional conservation of this protein in different species (Peiter et al., 2007a; Farthing et al., 2017; Ma et al., 2018). This is supported by a growth inhibition of *Osmtp11* knockdown mutants under Mn^2+^ toxicity, and an increased Mn^2+^ tolerance of *OsMTP11* overexpressor lines (Ma et al., 2018). In a parallel study, higher Mn^2+^ sensitivity of *Osmtp11* knockout lines was only apparent in the absence of *OsMTP8.1*, a vacuolar Mn^2+^ transporter (Tsunemitsu et al., 2018). As for Arabidopsis MTP11, localization of OsMTP11 is discrepant in different studies. It was found in the *trans*-Golgi (Farthing et al., 2017; Tsunemitsu et al., 2018) and the TGN, with a partial localization to the plasma membrane in *N. benthamiana* epidermal cells upon exposure to high extracellular Mn^2+^ concentration (Ma et al., 2018). Across all studies, the localization of all Golgi-bound Mn^2+^ transporters appears to vary and likely to depend on experimental conditions and/or cell types. A systemic analysis of this aspect is highly warranted.

Unlike in animals and fungi, a Ca^2+^ transporter accompanying the pump is not known yet. Also, the transport proteins identified so far have been associated with Mn^2+^ detoxification, whereas the Mn^2+^ supply of the complex Golgi-localized glycosylation processes is completely obscure. A role of ECA3 in glycosylation remains to be elucidated, and further transport proteins to support this function remain to be identified, whereby homologs of GDT1/TMEM165 represent promising candidates.

### Release of Ca^2+^ and Mn^2^^+^

In animal cells, the release of Ca^2+^ from the Golgi is mediated by RyRs and IP_3_Rs (Kellokumpu, 2019). In plants, there is no evidence of channels that take on this function. Also, [Ca^2+^]_Golgi_ in Arabidopsis is thought to be substantially lower than that in animals (Ordenes et al., 2012). It remains possible that this concentration is maintained by vesicular trafficking alone, but studies are required that monitor Ca^2+^ fluxes across vesicular membranes.

While a mechanism of Ca^2+^ release awaits discovery, a transporter that mediates the release of Mn^2+^ from the secretory pathway has recently been uncovered (Alejandro et al., 2017; Gao et al., 2018). The Arabidopsis NRAMP2 protein localizes to the TGN and rescues the low Mn^2+^-sensitive phenotype of the Δ*smf2* yeast mutant, which is devoid of the yeast NRAMP transporter SMF2 that mediates intracellular Mn^2+^ distribution in yeast. Growth of *nramp2* mutants was strikingly sensitive to Mn^2+^ deficiency (Alejandro et al., 2017; Gao et al., 2018), and in one study, expression of *NRAMP2* was induced in both shoots and roots under this stress (Alejandro et al., 2017). Defects in Mn^2+^ supply of chloroplasts and vacuoles suggest that NRAMP2 redistributes Mn^2+^ to these organelles, as discussed above (Alejandro et al., 2017). Moreover, NRAMP2 has been suggested to promote the reutilization of root-Mn^2+^, as Mn^2+^ concentrations were found to be higher in roots and lower in young leaves under Mn^2+^ starvation (Gao et al., 2018). However, since in another study, *nramp2* shoots accumulated more Mn^2+^ than those of the wild-type (Alejandro et al., 2017), this aspect demands further scrutiny.

## Ca^2+^ and Mn^2+^ in the vacuole

### Functions of Ca^2+^

Due to its tremendous size, the central vacuole represents the quantitatively most important organelle for storage and detoxification of metabolites and ions, including Ca^2+^ and Mn^2+^. Free Ca^2+^ in the vacuole is in the micro to millimolar range, but this parameter has been little studied. Owing to the steep electrochemical gradient of Ca^2+^ from the vacuole to the cytosol, the opening of cation channels in the tonoplast can rapidly increase [Ca^2+^]_cyt_ and thus contribute to cytosolic Ca^2+^ signaling (Peiter, 2011). However, while extracellular Ca^2+^ chelators make it relatively straightforward to discriminate between the apoplast and intracellular stores as source of [Ca^2+^]_cyt_ signals, it is less trivial to pinpoint the vacuole as intracellular source. The identification of the vacuolar channel protein TPC1, discussed below, eventually allowed to assign the vacuole a role in the generation of systemic Ca^2+^ signals in response to salt stress (Choi et al., 2014), herbivory and wounding (Kiep et al., 2015), and aphid feeding (Vincent et al., 2017).

Besides contributing to cytosolic Ca^2+^ signaling, luminal Ca^2+^ regulates vacuolar proteins either by direct binding or in an indirect way. The former is exemplified by TPC1 that has an inhibitory Ca^2+^-binding site on the luminal side, as discussed below (Dadacz-Narloch et al., 2011). Indirect regulation by luminal Ca^2+^ is assumed for a Na^+^ transporter that interacts with and is regulated by a vacuolar Calmodulin-like protein (Yamaguchi et al., 2005). However, the physiological relevance of this mechanism is unclear.

Release of vacuolar Ca^2+^ will first lead to a local increase in [Ca^2+^]_cyt_ at the tonoplast. This may be perceived again either directly or indirectly. Vacuolar channels, for example, TPC1 (Peiter et al., 2005), two-pore potassium 1 (TPK1; Gobert et al., 2007), and TPK3 (Höhner et al., 2019; Jaslan et al., 2019) are activated by Ca^2+^-binding EF-hand domains. Indirectly, autoinhibited Ca^2+^-ATPases in the tonoplast are activated by binding Ca^2+^-calmodulin (see below). CBL proteins together with CBL-interacting protein kinases (CIPKs) form modules to transduce Ca^2+^ signal information into phosphorylation events. In Arabidopsis, four CBLs (CBL2, 3, 6, and 10) localize to the tonoplast (Batistic et al., 2010) and may thus specifically sense Ca^2+^ released from the vacuole.

### Functions of Mn^2+^

Vacuoles play an important role in both detoxification and storage of Mn^2+^ in plant cells. Under Mn^2+^ excess, the sequestration of Mn^2+^ in vacuoles is a tolerance mechanism to avoid toxic effects in the cytosol. This mechanism is very obvious in Mn^2+^-(hyper)accumulating species that store vacuolar Mn^2+^ mainly in complex with carboxylates, such as malate, citrate, or oxalate (Dou et al., 2009; Fernando et al., 2010; Blamey et al., 2015).

Vacuolar Mn^2+^ sequestration is also activated under nutritional circumstances that lead to increased Mn^2+^ influx. Phosphate-efficient plants, such as lupins, mobilize phosphate by releasing large amounts of carboxylates into the rhizosphere (Lambers et al., 2015). This leads to the concurrent mobilization of Mn^2+^ that is massively taken up and deposited in leaf vacuoles (Peiter et al., 2000; Blamey et al., 2015). The transporter mediating this sequestration is unknown.

Iron deficiency is another nutritional imbalance that leads to critical Mn^2+^ influx. In Fe^2+^-limited dicotyledonous plants, Fe^2+^ is acquired by the poorly selective transporter IRT1. Mn^2+^ taken up by IRT1 has the potential to inhibit Fe(III) reduction mediated by ferric chelate reductase (FRO2), hence triggering iron chlorosis (Eroglu et al., 2016). In Arabidopsis, this problem is circumvented by sequestration of Mn^2+^ in root vacuoles by the vacuolar transporter MTP8 (Eroglu et al., 2016). Consequently, *mtp8* mutants are prone to iron chlorosis upon iron limitation in the presence of Mn^2+^. Vacuolar Mn^2+^ accumulation by MTP8 also protects imbibing seeds from Mn^2+^ toxicity (Eroglu et al., 2017). In cold temperatures, seeds can undergo numerous wet-and-dry cycles that mobilize soil Mn^2+^ by alternating reductive and oxidative conditions. This Mn^2+^ load is critical if not sequestered in the vacuole (Eroglu et al., 2017).

**Figure 3:**
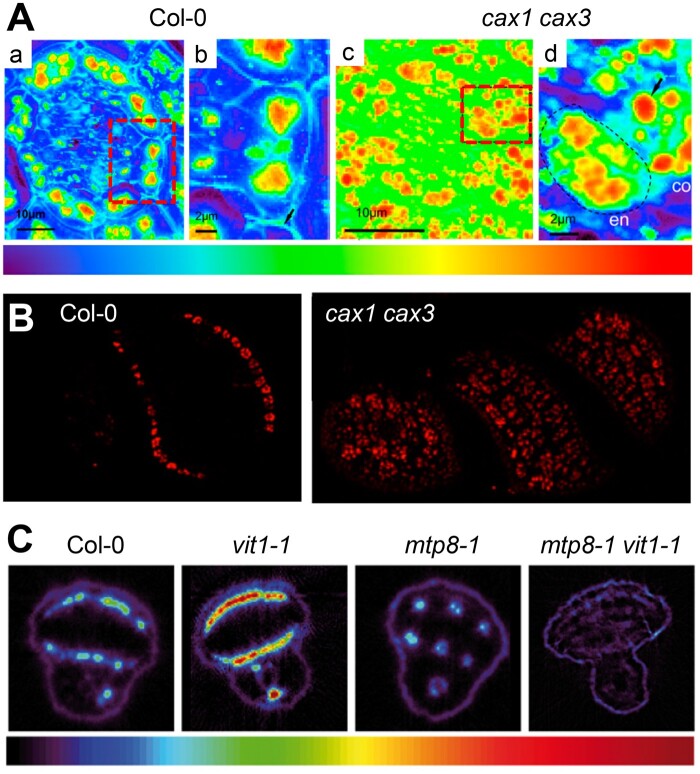
Synchrotron µXRF analyses visualize the allocation of Ca^2+^ and Mn^2+^ in Arabidopsis seeds by vacuolar transporters. A, High-resolution SXRF maps of Ca^2+^ distribution in endodermal layers of the hypocotyl (a and c) and single endodermal cells (b and d; positions indicated by rectangles in a and c, respectively) in seeds from Col-0 and *cax1cax3* plants. Colors indicate normalized fluorescence (logarithmic scale). Figure taken from Punshon et al. (2012), modified. B, High-resolution SXRF maps of Mn distribution in whole seeds of Col-0 and *cax1cax3* plants. Figure taken from Punshon et al. (2012), modified. C, Distribution of Mn in intact seeds from Col-0, *vit1-1*, *mtp8-1*, and *mtp8-1vit1-1* plants determined by µSXRF tomography. The color scale ranges from 0 to 1,100 µg g^−1^ Mn. Figure taken from Eroglu et al. (2017), modified.

Apart from detoxification, vacuolar Mn^2+^ serves as storage pool. Under Mn^2+^-limited conditions, vacuolar Mn^2+^ can thus be re-distributed to chloroplasts, which requires NRAMP3 and 4 (Lanquar et al., 2010; Alejandro et al., 2017). In the embryo of developing Arabidopsis seeds, Mn^2+^ is allocated specifically in the subepidermal cell layer on the abaxial side of the cotyledons and in the cortex of the hypocotyl, whereby MTP8 mediates the import into protein storage vacuoles of those cells (Chu et al., 2017; Eroglu et al., 2017; Figure 3). The physiological importance of this vacuolar Mn^2+^ storage pattern is reflected in poor germination of *mtp8* mutant seeds derived from Mn^2+^-limited mother plants (Eroglu et al., 2017). In the absence of MTP8, Mn^2+^ is allocated to the prevascular bundles by VIT1 (Figure 3).

### Import of Ca^2+^ and Mn^2+^

Ca^2+^ and Mn^2+^ are loaded into the vacuole by secondary active transporters, which employ the proton motive force (pmf) across the tonoplast, and by ACAs, of which ACA11 resides in the membrane of lytic vacuoles (Lee et al., 2007) and ACA4 in smaller vacuolar vesicles of Arabidopsis (Geisler et al., 2000). Knockout of both pumps causes the formation of lesions due to hypersensitive response, which is dependent on salicylic acid signaling (Boursiac et al., 2010). This phenotype has recently been related to an aberrant [Ca^2+^]_cyt_ regulation in *aca4aca11* plants. In the double mutant, basal [Ca^2+^]_cyt_ as well as flg22-induced Ca^2+^ signals in leaves were markedly increased (Hilleary et al., 2020). Both lesion formation and flg22-triggered Ca^2+^ signals were suppressed at elevated temperatures, which confirms the role of those tonoplast Ca^2+^ pumps as negative regulators of PAMP-triggered Ca^2+^ signals and innate immunity. In a separate study, the [Ca^2+^]_cyt_ response of root tips to external ATP was only minimally higher in *aca4aca11* as compared to the wild-type (Behera et al., 2018). The different outcomes point to a stimulus- or tissue-specific role of vacuolar ACAs. Transport of Mn^2+^ by those proteins has not been reported yet.

The molecular basis of Ca^2+^/H^+^ antiport across the tonoplast was uncovered with the identification of Arabidopsis CAX1 and CAX2 (Hirschi et al., 1996), which are regulated by an N-terminal autoinhibitory domain (Pittman et al., 2002). It has later become apparent that antiporters of the CAX family vary in substrate spectrum. CAX1 and CAX3 are selective for Ca^2+^, whereas CAX2 and CAX5 transport both Ca^2+^ and Mn^2+^ (Schaaf et al., 2002; Pittman and Hirschi, 2016).

Various physiological circumstances in which CAX proteins determine allocation and functions of both elements have been described. Overexpression of Arabidopsis *CAX1* in tobacco increased Ca^2+^ accumulation while causing symptoms of Ca^2+^ deficiency and defects in stress responses involving Ca^2+^ signaling (Hirschi, 1999). In contrast, *CAX1* knockout causes no strong growth phenotype, likely due to an ectopic upregulation of *CAX3* in the mutants (Cheng et al., 2003). Hence, double knockout plants for both genes are severely perturbed in Ca^2+^ distribution and ionome, triggering a host of physiological aberrations (Cheng et al., 2005; Conn et al., 2011). In dicots, including Arabidopsis, most Ca^2+^ is accumulated in vacuoles of mesophyll cells (Storey and Leigh, 2004), in which *CAX1* is most highly expressed. The *cax1cax3* mutant accumulates less Ca^2+^ in mesophyll vacuoles and contains higher Ca^2+^ concentrations in the apoplast. This leads to a reduced cell wall extensibility going along with transcriptional alterations of genes involved in cell wall modifications, and to defects in stomatal opening that again causes decreased CO_2_ assimilation (Conn et al., 2011). In contrast to dicots, in graminaceous plants, *CAX1* is mainly expressed in the epidermis (Conn and Gilliham, 2010), which again corresponds to the preferential accumulation of Ca^2+^ in this tissue (Karley et al., 2000).

The absence of *CAX1* alone and in combination with *CAX3* has a strong effect on the ionome, that is, the plant’s mineral element composition. Notably, Mn concentrations in shoots are reduced in *cax1*, while they are increased in *cax1cax3*, whereas Ca^2+^ and Mg^2+^ are decreased in the double mutant (Cheng et al., 2005). In addition, the deletion of CAX transporters reduces vacuolar H^+^-ATPase activity, which exerts numerous secondary effects.

CAX1 has been identified as a negative factor for adaptation to serpentine soils, which have a very high Mg^2+^:Ca^2+^ ratio, besides often high concentrations of heavy metals and a lack of nitrogen, phosphorus, and potassium (Bradshaw, 2005). Serpentine conditions are lethal to wild-type Arabidopsis, whereas *cax1* mutants are resistant to very high Mg^2+^ concentrations and high Mg^2+^:Ca^2+^ ratio, what has been explained by an increased cytosolic Ca^2+^ availability caused by the decreased sequestration (Bradshaw, 2005; Cheng et al., 2005).

*CAX1* and *CAX3* are both expressed during seed development, and SXRF analyses showed that their deletion disrupted the allocation of Ca^2+^ to organelles of the embryo (Punshon et al., 2012; Figure 3). Accordingly, deletion of *CAX*s negatively affects seed germination (Connorton et al., 2012). Distribution of Mn^2+^ is also disturbed in *cax1cax3* seeds (Punshon et al., 2012) although both transporters are selective for Ca^2+^ (Figure 3).

*CAX1* and *CAX3* are also co-expressed in guard cells and during stress responses, and both proteins physically interact, forming a transporter with altered kinetics (Zhao et al., 2009; Hocking et al., 2017). The functional relevance of CAX heteromers in stomatal regulation and in stress responses has been inferred from mutant analyses (Hocking et al., 2017).

CAX2 appears not to play a major role in Ca^2+^ homeostasis, but mediates vacuolar Mn^2+^ sequestration (Pittman et al., 2004). A three-amino acid region has been identified as instrumental for the Mn^2+^ specificity of this transporter (Shigaki et al., 2003). However, albeit its overexpression in tobacco caused an increased Mn^2+^ tolerance (Hirschi et al., 2000), sensitivity to Mn^2+^ was initially reported to be unaffected (Pittman et al., 2004), but later shown to be increased (Connorton et al., 2012) in a *cax2* knockout mutant. This discrepancy indicates that this transporter is not generally important for Mn^2+^ detoxification. CAX5 also transports Ca^2+^ and Mn^2+^, but had a lower transport velocity for both elements than its close relative CAX2 and also differs in expression pattern, with only *CAX5* being upregulated by elevated levels of Mn^2+^ (Edmond et al., 2009). Further work is required to examine a potential functional redundancy or co-operation of CAX2 and CAX5.

The vacuolar Ca^2+^ transporter CAX4 is transcriptionally upregulated by Mn^2+^ stress and Ca^2+^ depletion, and a *cax4* knockout mutant is Mn^2+^-sensitive (Cheng et al., 2002; Mei et al., 2009), making it likely that CAX4 is involved in the tolerance to Mn^2+^ toxicity. In this respect, the resistance of *cax1* mutants to Mn^2+^ toxicity and their altered Mn^2+^ accumulation may be the result of an induction of *CAX4* expression (Cheng et al., 2003). In addition, auxin responses of roots were altered in the *cax4* mutant (Mei et al., 2009). Mechanistic explanation of this phenotype may lie in the involvement of Ca^2+^ homeostasis in auxin signaling (Dindas et al., 2018) or in the role of Mn^2+^ in auxin conjugation (see above). Auxin responses were also affected in *cax1* and *cax3* mutants, causing a defective regulation of stomatal guard cells (Cho et al., 2012). This was explained by an increased apoplastic pH in the mutants, leading to a decrease in polar auxin transport. It remains to be shown if the auxin-related phenotypes of *cax1/3* and *cax4* are mechanistically related.

Orthologs of Arabidopsis CAXs have been described to reside in the vacuole of other species, such as VCAX1 in mung bean (Ueoka-Nakanishi et al., 2000) or VvCAX3 in grapevine (Martins et al., 2017). Albeit closely related to Arabidopsis CAX1/3, the latter also appears to transport Mn^2+^. Upon heterologous expression in yeast, OsCAX1a/b/c, 3, and 4 from rice confer Ca^2+^ tolerance, and OsCAX1a, 3, and 4 also Mn^2+^ tolerance, indicating that their substrate spectrum does not closely reflect that of their closest homologs in Arabidopsis (Kamiya et al., 2005; Yamada et al., 2014). *OsCAX1a* is expressed most highly and further induced by high Ca^2+^ levels (Kamiya et al., 2006), which suggests that it plays a crucial role in Ca^2+^ homeostasis similar to CAX1, its closest homolog in Arabidopsis. However, further studies using multiple mutants are required to dissect the relevance of CAXs in Mn^2+^ and Ca^2+^ homeostasis of rice.

Examination of homologs of Arabidopsis CAXs in other species revealed a phylogenetic diversity in their regulation. HvCAX2 of barley is apparently not regulated by an N-terminal domain, whereas in LeCAX2 of tomato the N-terminus only affects Mn^2+^ transport (Edmond et al., 2009). The structural basis as well as physiological consequences of those differences are still unknown.

To date, transporters of the CDF family have been described to mediate most physiological roles of vacuolar Mn^2+^ sequestration. As described above, in Arabidopsis, expression of MTP8 is highly induced by Fe deficiency, but also by high Mn^2+^ levels (Eroglu et al., 2016), and this transporter additionally builds vacuolar Mn^2+^ pools in specific embryo tissues (Chu et al., 2017; Eroglu et al., 2017). Similar to Arabidopsis, the rice transporters OsMTP8.1 and OsMTP8.2 mediate Mn^2+^ tolerance through sequestration of Mn^2+^ into vacuoles (Chen et al., 2013; Takemoto et al., 2017). The founding member of the Mn^2+^-transporting CDF subfamily, *Stylosanthes hamata* MTP8, transports Mn^2+^ upon expression in yeast. Its overexpression in Arabidopsis conferred Mn^2+^ tolerance (Delhaize et al., 2003), but its function in *S. hamata* has yet to be confirmed.

Proteins of the VIT family, such as Arabidopsis VIT1, have also been described as vacuolar Mn^2+^ transporters, albeit the primary function of VIT1 is to move Fe^2+^ into vacuoles of developing embryos (Kim et al., 2006). Similarly, OsVIT1 and OsVIT2 are vacuolar Mn^2+^ transporters, but their likely main function in rice is to mediate the vacuolar sequestration of Fe^2+^ and Zn^2+^ in flag leaves for future transport to the seeds (Zhang et al., 2012b). In wheat, ectopic expression of the ortholog *TaVIT2* in the endosperm caused a higher accumulation of Mn^2+^ and Fe^2+^, which was suggested as biofortification strategy (Connorton et al., 2017). In general, Mn^2+^ appears to be a secondary substrate of VIT proteins.

### Release of Ca^2+^ and Mn^2+^

Our mechanistic understanding of vacuolar Ca^2+^ release is still rudimentary. In fungi, this is mediated by proteins of the transient receptor potential (TRP) family (Lange et al., 2016), which are absent in plants. Flux analyses and patch-clamp studies on vacuolar membranes of various plant species have demonstrated the presence of Ca^2+^ conductances activated by IP_3_ or cADPR (see Peiter, 2011 for review). In animals, these ligands activate IP_3_Rs and RyRs, respectively, as discussed above for the ER and the Golgi. As plants lack those protein families (Edel et al., 2017), the mode of action of those molecules is still unclear. Their effectiveness on isolated vacuolar membranes suggests a direct action on channels or associated proteins, which is supported by the binding of IP_3_ to the vacuolar membrane with high-affinity (Brosnan and Sanders, 1993). However, later it was shown that IP_6_ is a far more effective agonist of Ca^2+^ release, and that IP_3_ is rapidly converted to IP_6_ (Lemtiri-Chlieh et al., 2003). We are still awaiting the breakthrough that solves the enigma of IP_x_-mediated Ca^2+^ signaling in plants.

The slow vacuolar (SV) channel, encoded by *TPC1*, has emerged as one of the regulators of [Ca^2+^]_cyt_. Identified electrophysiologically in the 1980s (Hedrich et al., 1986), it represents the longest-known and most extensively scrutinized ion channel in plants (Hedrich and Marten, 2011; Hedrich et al., 2018; Pottosin and Dobrovinskaya, 2018). Its molecular identity was revealed 20 years later (Peiter et al., 2005). Studies of SV currents have unearthed a plethora of factors that regulate this channel in vitro, including membrane potential, redox potential, phosphorylation, pH, Ca^2+^, Mg^2+^, polyamines, and 14-3-3 proteins (Peiter, 2011). These factors do not act independently on the channel, and some have opposite effects on either side of the membrane, which adds further complexity. Albeit tempting, an amalgamation of these studies is difficult as they have been conducted on different species and tissues and with different experimental set-ups.

The TPC1 protein, which functions as dimer, contains two Shaker-like structures, each containing six transmembrane spans and a pore domain, fused by a cytosolic linker. The structure of Arabidopsis TPC1 has been solved in independent labs with high resolution (Guo et al., 2016; Kintzer and Stroud, 2016), which allows to elucidate the molecular basis of its electrophysiological characteristics (Kintzer and Stroud, 2018). In animals, TPC channels are the molecular basis of NAADP-activated Ca^2+^ release from lysosomes and endosomes (see Jin et al., 2020 for review), while other studies found them to be Na^+^-selective and activated by the phosphoinositide PI(3,5)P_2_ (Wang et al., 2012). Intriguingly, recent research indicated that, depending on the ligand, animal TPC channels show different selectivities toward Ca^2+^ or Na^+^ (reviewed in Gerndt et al., 2020). Arabidopsis TPC1 is neither sensitive to NAADP nor to PI(3,5)P_2_ (Boccaccio et al., 2014).

It is now commonly accepted that TPC1 resides exclusively in the vacuolar membrane of mono- and dicotyledonous plants (Peiter et al., 2005; Larisch et al., 2012; Dadacz-Narloch et al., 2013). It conducts mono- and divalent cations, including Ca^2+^. Nevertheless, it is still controversially debated if TPC1 is able to function as a bona fide Ca^2+^ release channel (Hedrich et al., 2018). It has been discovered early that TPC1 currents are activated by increases in [Ca^2+^]_cyt_ (Hedrich and Neher, 1987), which is owing to the presence of two EF-hands in the cytosolic linker, of which only EF2 appears to be involved in Ca^2+^ activation (Schulze et al., 2011). This feature would allow TPC1 to function as CICR channel (Ward and Schroeder, 1994; Bewell et al., 1999). Patch-clamp measurements of ion currents combined with [Ca^2+^] determination by a fluorescent reporter directly demonstrated the dependence of Ca^2+^ fluxes across the vacuolar membrane on TPC1 (Gradogna et al., 2009; Carpaneto and Gradogna, 2018). However, unphysiologically positive voltages required to activate TPC1 and its inhibition by luminal Ca^2+^ have raised doubts about its role in Ca^2+^ release (Rienmüller et al., 2010). The structural basis of this inhibition by luminal Ca^2+^ lies in a Ca^2+^-binding domain on the luminal side (Dadacz-Narloch et al., 2011). Defunctionalization of this domain in the *fou2* mutant results in a hyperactive TPC1 that leads to constitutive JA signaling (Bonaventure et al., 2007; Beyhl et al., 2009), which is thought to be caused by an altered tonoplast potential (Lenglet et al., 2017).

It was recently shown that TPC1, together with TPK1 and TPK3, is a prerequisite for vacuolar excitability (Jaslan et al., 2019). Mutant analyses have also pointed to roles of TPC1 in stomatal regulation and seed germination (Peiter et al., 2005). Two studies have shown that TPC1 is a target of selection on serpentine soils, which have a very low Ca^2+^ versus Mg^2+^ availability (Turner et al., 2010; Arnold et al., 2016). *Arabidopsis arenosa* and *A. lyrata* populations adapted to those extreme conditions have a severely altered ionome with a very high Ca^2+^/Mg^2+^ ratio, which coincides with alterations in several genes associated with ion transport and homeostasis (Arnold et al., 2016). These comprise a non-synonymous mutation in the pore of TPC1, which may provide a mechanistic basis of the altered Ca^2+^ and Mg^2+^ handling or of changes in Ca^2+^ signaling in serpentine-adapted plants.

Attempts to assign a role in Ca^2+^ signal generation to this channel had initially failed (Ranf et al., 2008). This changed when the propagation of systemic [Ca^2+^]_cyt_ signals evoked by salt stress, wounding, and aphid feeding was demonstrated to require TPC1. In a *tpc1* mutant, a NaCl-triggered Ca^2+^ wave and local-systemic Ca^2+^ signals caused by aphids are dampened (Choi et al., 2014; Vincent et al., 2017), while a wounding-induced Ca^2+^ wave is even abolished (Kiep et al., 2015). A recent study suggested that [Ca^2+^]_cyt_ homeostasis in systemic signaling is related to the TPC1/TPK1/TPK3-dependent excitability of the vacuolar membrane (Dindas et al., 2021). In this model, which deserves further scrutiny, TPC1 as part of a Ca^2+^-homeostat plays an indirect role in [Ca^2+^]_cyt_ control by determining H^+^/Ca^2+^ antiport across the vacuolar membrane. The specific involvement of TPC1 in systemic [Ca^2+^]_cyt_ signal generation provokes the question of which factors regulate its activity in vivo.

Recently, a member of the GLR family, GLR3.6, was suggested to be localized to vacuolar membranes in xylem contact cells (Nguyen et al., 2018). Mutation of this channel together with that of GLR3.3 (see above) attenuated or abolished wounding-induced membrane potential changes and Ca^2+^ waves (Mousavi et al., 2013; Vincent et al., 2017; Nguyen et al., 2018; Shao et al., 2020). Ca^2+^ permeability of GLR3.6 has been demonstrated by expression in HEK293T cells (Shao et al., 2020). Similar to GLR3.3 and 3.1, most evidence points to a function of GLR3.6 in the plasma membrane (Mou et al., 2020). The subcellular localization of those channels thus requires further scrutiny.

A further potential mechanism of vacuolar Ca^2+^ release may rely on annexins, that have been localized to the tonoplast besides the plasma membrane (Seals et al., 1994) and shown to permeate Ca^2+^ in lipid bilayer experiments (Laohavisit et al., 2012). Annexin-mediated Ca^2+^ currents are activated by reactive oxygen species (ROS), which would allow them to contribute to a ROS wave-driven Ca^2+^ wave (Richards et al., 2014), and, accordingly, Annexin1 is required for the propagation of systemic Ca^2+^ signals (Malabarba et al., 2021). The contribution of annexins to the generation of [Ca^2+^]_cyt_ signals demands further attention.

It has not been studied yet if the potential vacuolar Ca^2+^ release channels also conduct Mn^2+^. For TPC1, this is highly expected, since it is permeable to Mg^2+^ in a hydrated state (Pottosin and Dobrovinskaya, 2018), but this awaits to be studied. Transporters that move vacuolar Mn^2+^ into the cytosol have been identified. ZIP1 of the ZRT family is a transporter localized in root vacuoles and likely involved in remobilizing Mn^2+^, but its function in cellular Mn^2+^ homeostasis is still unknown (Milner et al., 2013). Proteins of the NRAMP family, Arabidopsis NRAMP3 and 4, as well as *Thlaspi caerulescens* TcNRAMP3 and 4, also remobilize vacuolar Mn^2+^ (Thomine et al., 2003; Oomen et al., 2009). Mutation of *NRAMP3* and *4* renders the plant hypersensitive to Mn^2+^ deficiency, which is associated with a decrease in chloroplastic Mn^2+^ and reduced photosynthetic activity (Lanquar et al., 2010). This indicates an important role of these vacuolar Mn^2+^ efflux transporters for Mn^2+^ loading into the chloroplasts. A mechanistic explanation of the distribution of released vacuolar Mn^2+^ remains to be established, and possibly involves vesicular trafficking, as discussed above.

## Ca^2+^ and Mn^2+^ in the chloroplast

### Functions of Ca^2+^

The chloroplast is the site of photosynthesis and many biosynthetic pathways, and it accumulates high concentrations of Ca^2+^ and Mn^2+^ (Finazzi et al., 2015). Both cations play important roles in chloroplast structure and functions. One Ca and four Mn atoms are present in the Mn_4_CaO_5_ cluster in the OEC of PSII, which oxidizes H_2_O to provide electrons needed in the photosynthetic electron transport chain and protons to generate ATP (Umena et al., 2011). While Mn catalyzes the oxidation of H_2_O, the Ca^2+^ modulates the redox potential of the OEC to allow Mn to transfer electrons away from the O_2_ (Tsui et al., 2013). A redox-active tyrosine is oxidized and reduced in a proton-coupled electron transfer reaction, which is dependent on Ca^2+^ (Guo et al., 2018). Furthermore, Ca^2+^ is capable of binding to PsbO, an extrinsic protein of the PSII complex (Murray and Barber, 2006).

Ca^2+^ regulates many proteins involved in photosynthetic reactions, organelle division, and the chloroplastic protein import machinery (Rocha and Vothknecht, 2012; Stael et al., 2012; Hochmal et al., 2015). The ion may be an important regulator of several key enzymes of the photosynthetic light-independent reaction, the Calvin–Benson–Bassham cycle, including fructose 1,6-bisphosphatase and sedoheptulose 1,7-bisphosphatase (Charles and Halliwell, 1980; Kreimer et al., 1988). Furthermore, the import of nuclear-encoded proteins via the translocon at the inner envelope membrane of chloroplasts (TIC) complexes may be under the regulation of Ca^2+^. Calmodulin directly binds to the stromal side of TIC32, whereas Ca^2+^ itself has a strong effect on TIC110 channel activity in vitro (Chigri et al., 2006; Balsera et al., 2009). An important target of Ca^2+^ in the chloroplast is the Ca^2+^-sensing receptor (CAS), a thylakoid membrane-associated Ca^2+^-binding protein that has been identified as a central regulator of cellular Ca^2+^ homeostasis and signaling. CAS modulates chloroplast stromal Ca^2+^ signals induced by darkness, flg22, and heat, and, surprisingly, it governs external-Ca^2+^-induced Ca^2+^ transients in the cytosol (Nomura et al., 2008; Weinl et al., 2008). The protein regulates photosynthetic efficiency, modulates MAPK signaling, and participates in plant stress responses, where it plays a role in stomatal regulation and innate immunity (Vainonen et al., 2008; Weinl et al., 2008; Nomura et al., 2012). The mechanistic basis of CAS action is poorly understood. Finally, the vacuolar Ca^2+^-activated K^+^ channel TPK3 (see above), has also been described to localize to thylakoid membranes and to be vital for the generation of the pmf (Carraretto et al., 2013). However, both localization of TPK3 in chloroplasts and its involvement in photosynthetic processes have recently been questioned (Höhner et al., 2019). The reason for the diametrically opposed findings in those studies remains to be determined.

The regulatory role of Ca^2+^ in the chloroplast implies dynamic fluctuations of free Ca^2+^ in its subcompartments. Changes in [Ca^2+^]_stroma_ with different kinetics are indeed induced by numerous biotic (flg22, chitin, cryptogein, and oligogalacturonides) and abiotic (osmotic, heat, cold, oxidative, and salt) stresses as well as environmental stimuli (light-to-dark transition; Navazio et al., 2020). [Ca^2+^]_stroma_ signals in response to stress occur in chloroplasts and nongreen plastids, whereas the response to light-dark transition is chloroplast-specific, suggesting a potential role of the thylakoids in its generation (Sello et al., 2016). Imaging of individual chloroplasts revealed the occurrence of [Ca^2+^]_stroma_ spiking to blue pulsed light, in addition to the previously described sustained transient (Loro et al., 2016). The [Ca^2+^]_stroma_ transient at the onset of darkness is hypothesized to initiate an alteration of metabolic processes, as recently shown for ppGpp synthesis (Ono et al., 2020). It is not accompanied by a decrease in [Ca^2+^]_cyt_, which suggests that it is generated by Ca^2+^ release from an intrachloroplastic store (Sai and Johnson, 2002). This store might be the thylakoid lumen, in which free Ca^2+^ is 3–5 times higher than in the stroma (Sello et al., 2018). However, [Ca^2+^]_thylakoid_ does not decrease reciprocally to the transient rise in [Ca^2+^]_stroma_, but rather shows a steady increase (Sello et al., 2018). Alternatively, Ca^2+^ may be released from negatively charged thylakoid membranes or Ca^2+^-binding proteins, to which it can be reversibly bound.

Intriguingly, the dark-induced [Ca^2+^]_stroma_ transient has recently been demonstrated to be accompanied by a transient *increase* in [Ca^2+^]_cyt_. Both the [Ca^2+^]_cyt_ and the [Ca^2+^]_stroma_ response are gated by the circadian clock and reach maximum levels at anticipated dusk (Ruiz et al., 2020). These findings suggest that the circadian clock determines chloroplast function by gating [Ca^2+^]_stroma_ signals. It furthermore opens the question if both [Ca^2+^] elevations are mechanistically linked, for example, by dark-induced Ca^2+^ release from the chloroplast.

Recently, elevated temperatures ˃30°C were shown to cause a [Ca^2+^]_stroma_ elevation dependent on absolute temperature, rather than the rate of heating (Lenzoni and Knight, 2019). This Ca^2+^ response involves the CAS protein. It is specific to the chloroplast and is thereby clearly distinguished from a Ca^2+^ signal elicited by stronger heat, which occurs in the cytosol by influx of apoplastic Ca^2+^ (Finka et al., 2012).

Taken together, Ca^2+^ dynamics in the chloroplast are initiated by different stimuli. They likely have many regulatory roles in the organelle and influence the entire cellular signaling network. However, our mechanistic understanding of the physiological consequences of plastidial Ca^2+^ signals is still very incomplete.

### Functions of Mn^2+^

As the catalyst in the OEC of PSII, Mn^2+^ takes center stage in photosynthesis. Therefore, Mn^2+^ deficiency strongly affects this process, leading to reduced Mn^2+^ binding to PSII, which causes the destabilization of its super and subcomplexes (Schmidt et al., 2016; 2020). PsbP is one of the extrinsic proteins and has two Mn^2+^-binding sites, suggesting PsbP may function as a Mn^2+^ carrier that facilitates the access of Mn^2+^ ions to the OEC (Cao et al., 2015). Moreover, the phosphatase TAP38/PPH1, which specifically targets light-harvesting complex II, contains a Mn^2+^ (or Mg^2+^) binuclear center, which unravels a crucial step in state transitions between PSI and PSII (Wei et al., 2015).

In addition to photosynthesis, Mn^2+^ is required by enzymes in biosynthetic pathways. This includes imidazoleglycerol-phosphate dehydratase involved in histidine biosynthesis (Bisson et al., 2015), as well as 3-deoxy-D-arabino-heptulosonate 7-phosphate synthase, which is the first enzyme of the Shikimate pathway for the production of chorismate, the precursor of aromatic amino acids and secondary metabolites (Entus et al., 2002). Finally, the catalytic activity of glutamine synthetase is regulated by Mn^2+^ and Mg^2+^ (Eisenberg et al., 2000).

Several other enzymes, including phytoene synthetase, mevalonic kinase, and *ent*-kaurene synthetase in the isoprenoid biosynthetic pathway that produces building blocks of carotenoids, chlorophyll, and gibberellins, require Mn^2+^ as a cofactor (Dogbo et al., 1988; Rohdich et al., 2006). The decrease in the production of pigments and gibberellins under Mn^2+^ deficiency has been assigned to this function (Wilkinson and Ohki, 1988), what should be followed-up. Intriguingly, it is hypothesized that Mn^2+^ and Mg^2+^ function as toggle switch for some chloroplastic enzymes, including Rubisco, malic enzyme, and phosphoglycolate phosphatase, to improve the energy balance between photorespiration and other metabolic processes (Bloom and Lancaster, 2018).

### Import and allocation of Ca^2+^ and Mn^2+^

Three types of membranes (an inner and outer envelope and a thylakoid membrane) divide the chloroplast into three distinct internal compartments, consisting of the intermembrane space between the two chloroplast envelope membranes, the stroma, and the thylakoid lumen. To transport ions and metabolites through these three membranes, the chloroplast harbors transport proteins in each of them (Carraretto et al., 2016; Schmidt et al., 2020).

Early biochemical studies demonstrated that Ca^2+^ uptake from the cytosol into the chloroplast is a light-dependent process (e.g. Muto et al., 1982), and that Ca^2+^ is imported into the thylakoid lumen by Ca^2+^/H^+^ antiport (Ettinger et al., 1999). Some candidate proteins localized to the chloroplast of Arabidopsis were proposed to mediate Ca^2+^ flux across the chloroplast inner envelope. This includes a heavy-metal ATPase (Moreno et al., 2008) and the GLR channels GLR3.4 and GLR3.5 (Teardo et al., 2011; 2015), with additional biochemical evidence supporting GLR activity in chloroplast inner envelope of spinach (Teardo et al., 2010). However, none of these were demonstrated to alter [Ca^2+^] or mediate Ca^2+^ transport in intact chloroplasts, and GLR3.4 and GLR3.5 have also been reported to be localized in the plasma membrane (Meyerhoff et al., 2005; Teardo et al., 2011; Kong et al., 2016).

This situation has changed recently with the identification of chloroplast-targeted homologs of yeast GDT1 that contributes to Ca^2+^/Mn^2+^ homeostasis in the Golgi of yeast, as described above. These transporters were described independently by several groups as representing long-sought Mn^2+^ and Ca^2+^ pathways across chloroplast membranes and tagged with an array of nonsystematic names that may lead to confusion. Owing to its phenotype, a thylakoid-localized transporter was first described as photosynthesis-affected mutant71 (PAM71), with a second member named PAM71-homolog (PAM71-HL; Schneider et al., 2016). For its presumed transport function and localization, this protein was also named Chloroplast-localized Ca^2+^/H^+^ Antiporter 1 (CCHA1; Wang et al., 2016). Later, PAM-71-HL was renamed chloroplast Mn^2+^ transporter 1 (CMT1) based on one of its substrates and its localization (Eisenhut et al., 2018; Zhang et al., 2018). This situation was unsatisfactory because proteins of this family are neither all likely to be localized to chloroplasts, nor are they selective transporters for Ca^2+^ or Mn^2+^. In our analysis of those proteins, we, therefore, renamed them bivalent cation transporter1 (BICAT1) and BICAT2, based on the substrate spectrum of all known eukaryotic homologs (Frank et al., 2019). This nomenclature has found wide acceptance in the community (Stael, 2019; Navazio et al., 2020; Ruiz et al., 2020; Schmidt et al., 2020; Pirayesh et al., 2021).

BICAT1 is localized to the thylakoid, while BICAT2 resides in the inner envelope of the chloroplast (Schneider et al., 2016; Wang et al., 2016; Eisenhut et al., 2018; Zhang et al., 2018; Frank et al., 2019). The complementation of yeast mutants defective in Mn^2+^ and Ca^2+^ transport indicated the permeation of both cations by those proteins. Interestingly, two studies employing different mutants indicated that BICAT2 mediates Mn^2+^ influx into or Mn^2+^ efflux from the cytosol (Eisenhut et al., 2018; Zhang et al., 2018). Transport of Ca^2+^ by BICAT1 and BICAT2 was confirmed by transport assays employing *Escherichia coli* or yeast vesicles (Frank et al., 2019). In agreement with an activity in Ca^2+^ transport, mutants devoid of *BICAT1* or *BICAT2* were defective in ^45^Ca^2+^ uptake by illuminated isolated thylakoids and chloroplasts, respectively (Frank et al., 2019). Expectedly, the impeded Ca^2+^ movement impinges on Ca^2+^ homeostasis in the stroma, as detected by a stroma-targeted aequorin reporter: the [Ca^2+^]_stroma_ transient evoked by light-dark transition was almost completely abolished in *bicat2* mutants, but increased in *bicat1* mutants (Frank et al., 2019). Based on the presumed Ca^2+^ fluxes that contribute to this Ca^2+^ signal, it is conceivable that BICAT2 imports cytosolic Ca^2+^ during the light phase. Imported Ca^2+^ may be bound, as described above, and released to the stroma upon darkness. Stromal Ca^2+^ may subsequently be translocated by BICAT1 into thylakoids or released into the cytosol. This sequence is supported by the Ca^2+^ dynamics in cytosol and lumen (Sello et al., 2018; Ruiz et al., 2020) in combination with the effects of *BICAT* knockout (Frank et al., 2019). The kinetics of dark-induced [Ca^2+^]_stroma_ changes in a *bicat1bicat2* double mutant are indistinguishable from those of *bicat2* single mutants, as expected from a model where *bicat2* acts upstream of *bicat1* (Frank et al., 2019). The regulation of chloroplast Ca^2+^ homeostasis by BICAT proteins is bound to affect the many Ca^2+^-regulated processes described above, what remains to be studied. This is, however, complicated by the other essential function of those transporters, the transport of Mn^2+^ into the chloroplast and the lumen.

Mutants of *bicat2* are defective in Mn^2+^ accumulation in chloroplasts, and Mn^2+^ partitioning is altered in *bicat1* (Schneider et al., 2016; Eisenhut et al., 2018; Zhang et al., 2018). Consequently, Mn^2+^ binding to PSII complexes is reduced and the mutants are defective in PS II activity, which is most severe in *bicat2*. In *bicat1*, but not in *bicat2*, PSII activity was restored by supplementation of Mn^2+^ (Schneider et al., 2016; Eisenhut et al., 2018).

Phenotypically, *bicat2* mutants display severe defects in growth, chloroplast morphology, and photosynthetic activity, while *bicat1* mutants are less affected, and the *bicat1bicat2* phenotype is similar to that of *bicat2*. This highlights again the dominant role of BICAT2 in chloroplast structure and functions (Schneider et al., 2016; Eisenhut et al., 2018; Zhang et al., 2018; Frank et al., 2019). An ortholog of BICAT1 in *Zea mays ssp. mexicana* L., ZmmCCHA1, was recently described to be functionally comparable to its Arabidopsis counterpart, which indicates the conservation of these mechanisms in monocots (Wang et al., 2020).

Further investigations are required to unravel the transport mode and selectivity of chloroplast BICATs that determine their dual function. Their parallel interference in Ca^2+^ and Mn^2+^ homeostasis necessitates discriminative mechanisms and complicates the analysis of the role of those transporters in planta.

With the recent description of a chloroplast-localized mitochondrial Ca^2+^ uniporter (cMCU), a Ca^2+^ channel localized in the chloroplast inner envelope as well as in root mitochondria was identified (Teardo et al., 2019). By mediating the flux of Ca^2+^ into the chloroplast, it contributes to the elevation of [Ca^2+^]_stroma_ in response to oxidative and osmotic stress. In accordance with a role in osmotic (drought) stress responses, signaling events downstream of the Ca^2+^ signal were altered, and mutants were drought-tolerant (Teardo et al., 2019). This breakthrough provokes a host of new questions. Why does it only contribute to [Ca^2+^]_stroma_ elevations to some specific signals? What is its functional cooperation with BICAT2 in the same membrane? Owing to different affinities, they may operate at different ion concentrations (Frank et al., 2019; Stael, 2019). Finally, since mammalian MCU proteins are permeable to Mn^2+^ (Kamer et al., 2018), as discussed for mitochondria below, a further role of cMCU may lie in Mn^2+^ supply or release of chloroplasts, what remains to be studied.

## Ca^2+^ and Mn^2+^ in the mitochondrion

### Functions of Ca^2+^

Compared to chloroplasts, the role of Ca^2+^ in plant mitochondria has been less studied. Excellent reviews provide an overview of the subject (Stael et al., 2012; Wagner et al., 2016; Costa et al., 2018; Pirayesh et al., 2021). In animals, free mitochondrial Ca^2+^, [Ca^2+^]_mito_, is a central regulator of aerobic metabolism (Szabadkai and Duchen, 2008). This regulatory function is based on the parallel activation of Ca^2+^-dependent enzymes of the tricarboxylic acid (TCA) cycle, that is, pyruvate dehydrogenase (PDH), NAD^+^-isocitrate dehydrogenase (NAD-ICDH), and 2-oxoglutarate dehydrogenase (OGDH; McCormack et al., 1990). Regulation by Ca^2+^ is pursued by direct binding (NAD-ICDH, OGDH) or Ca^2+^-dependent dephosphorylation (PDH; Giacomello et al., 2007). The ATP synthase is also Ca^2+^-activated. Collectively, this regulates the production of NAD(P)H^+^ and ATP synthesis. Such a role of [Ca^2+^]_mito_ is less clear in plants.

A particular role of [Ca^2+^]_mito_ in animals is to trigger apoptotic cell death. Thereby the mitochondrial permeability transition pore, a membrane-spanning multi-protein complex, that likely originates from the F-ATP synthase, is activated by mitochondrial Ca^2+^ overaccumulation, causing membrane rupture and the release of apoptotic signals, such as cytochrome *c* (Giacomello et al., 2007; Carraro et al., 2019). This sequence of events is also found in plant mitochondria (Arpagaus et al., 2002; De Col et al., 2018), indicating a high degree of conservation.

A regulation by [Ca^2+^]_mito_ is to be expected for mitochondrial proteins that carry a Ca^2+^-binding EF hand. In plants, this includes NAD(H)-dependent glutamate dehydrogenase2 (GDH2), but not GDH1 (Turano et al., 1997). Both isoforms form homo- and hetero-hexamers in all combinations, hence assembling seven different isozymes with different total numbers of EF-hands. This may produce a spectrum of Ca^2+^ dependencies, which has indeed been observed in vitro in older studies (Loulakakis and Roubelakis-Angelakis, 1990). In plants, GDH operates primarily in the catabolic direction to release NH_4_^+^ and 2-oxoglutarate from glutamate. Intriguingly, Ca^2+^ appears to stimulate GDH only in the opposite, aminating direction, required to detoxify high amounts of NH_3_, which may occur in mitochondria during photorespiration or uptake of NH_4_^+^ from the soil (Tercé-Laforgue et al., 2004). [Ca^2+^]_mito_ may thus play a decisive role in GDH activation and fine-tuning, what remains to be demonstrated in vivo.

There are still numerous loose ends regarding the action of Ca^2+^ in plant mitochondria. Pharmacological studies indicated that the import of nuclear-encoded proteins into plant mitochondria across the inner membrane is Ca^2+^- and CaM-regulated (Kuhn et al., 2009). As yeast mitochondria are unaffected by the pharmacological treatments, this trait is suspected to be plant-specific, whereby the mechanism of action is unclear. Possibly related to this, a calmodulin-like protein, CML30, has been localized to mitochondria, but its role has not been revealed yet (Chigri et al., 2012).

The concentration of free Ca^2+^ in plant mitochondria is about twice than that in the cytosol and responsive to stimuli (Logan and Knight, 2003). The dynamics of [Ca^2+^]_mito_, that regulate the mitochondrial Ca^2+^-dependent processes, are linked to those in the cytosol. Generally, [Ca^2+^]_mito_ follows [Ca^2+^]_cyt_ elevations, but with specific kinetics (Loro et al., 2012). Mitochondria may thus act as sinks of cytosolic Ca^2+^ and in turn shape the spatiotemporal signature of cytosolic Ca^2+^ signals. This property has been demonstrated in animal cells (e.g. in cardiomyocytes, Drago et al., 2012), whereas in plants it remains to be shown.

### Functions of Mn^2+^

An important function of Mn^2+^ in mitochondria is as part of the active site of MnSOD, which removes superoxide anion radicals generated as a side-product of energy metabolism (Andresen et al., 2018). Decrease in MnSOD activity inhibits the TCA flux and affects the mitochondrial redox balance, causing a decrease in plant growth (Morgan et al., 2008). Although mitochondrial MnSOD function is reduced by Mn deficiency in Chlamydomonas (Allen et al., 2007), MnSOD activity in plants, as Arabidopsis, is unaffected under low Mn^2+^ supply (Lanquar et al., 2010; Alejandro et al., 2017). This indicates that organelles are prioritized differently in both organisms.

Arginase is another Mn^2+^-containing metalloenzyme in plant mitochondria, whose function is to hydrolyze arginine to ornithine and urea, which is recycled by urease to ammonia (Cao et al., 2010). Arabidopsis has two mitochondrial arginases, ARGAH1 and ARGAH2 (Flores et al., 2008), of which ARGAH2 was also localized in chloroplasts (Patel et al., 2017). A coordinated action of arginase and urease remobilizes nitrogen to meet the metabolic demands of developing seedlings (Winter et al., 2015). Single and double mutants show an increased nitric oxide (NO) accumulation, going along with altered auxin responses in roots (Flores et al., 2008) and increased abiotic stress resistance (Shi et al., 2013). NO production in plant mitochondria may be mediated by NO synthase (NOS)-like enzymes that convert arginine into NO and citrulline (Winter et al., 2015), besides a nitrate-dependent pathway (Planchet et al., 2005). As arginine is also a precursor of polyamines, arginase mutants have increased levels of putrescine and spermine (Shi et al., 2013). Polyamines may also be precursors of NO biosynthesis (Tun et al., 2006). Hence, Mn^2+^-containing arginases play distinctive roles in the crosstalk between arginine metabolism and the accumulation of polyamines and NO, resulting in a range of responses to abiotic stresses.

In animal cells, the main enzymes capable of generating reducing power (i.e. NADH) in mitochondria are the NAD-dependent malic enzymes (NAD-MEs), which catalyze the oxidative decarboxylation of malate to yield pyruvate and CO_2_, and the NAD-ICDHs that catalyze the oxidative decarboxylation of isocitrate to 2-oxoglutarate. Both require Mn^2+^ or Mg^2+^ for the catalysis (Yang et al., 2002; Ma et al., 2017). Intriguingly, NAD-ICDH is also activated by Ca^2+^, as mentioned above. Arabidopsis contains two genes encoding catalytic ICDH subunits and three genes encoding regulatory subunits (Lemaitre et al., 2007), as well as two NAD-ME proteins that form homodimers or a heterodimer with distinct functional characteristics (Tronconi et al., 2010). Their activity has been analyzed in vitro in the presence of Mn^2+^ (Lemaitre et al., 2007; Tronconi et al., 2010) but the importance of this metal and an interference with Ca^2+^ in the regulation of their activity in vivo requires further investigation.

### Import of Ca^2+^ and Mn^2+^

Due to the high membrane potential of around −180 mV across the inner mitochondrial membrane (IMM), which is due to the activity of the electron transport chain, Ca^2+^ would accumulate to 100–200 mM in the mitochondrial matrix if it was in Nernstian equilibrium. This would of course be detrimental. In mammalian mitochondria, Ca^2+^ accumulates to only 1–10 µM in the matrix, implying the operation of regulated influx pathways and efflux mechanisms (Szabadkai and Duchen, 2008). The outer mitochondrial membrane (OMM) is mostly regarded as unselectively permeable, not restricting Ca^2+^ import (Wagner et al., 2016). Conversely, Ca^2+^ influx across the inner membrane of animal mitochondria is mediated by a Ca^2+^-selective channel, the MCU (Kirichok et al., 2004). The channel protein responsible for those Ca^2+^ currents contains only two transmembrane domains and a short pore loop (Baughman et al., 2011; De Stefani et al., 2011). Its structure has been solved, revealing the basis of its high ion selectivity (Baradaran et al., 2018). Notably, its absence abrogates Ca^2+^ uptake by animal mitochondria, whereas there is not yet a clear in vivo demonstration that MCU is the principal mechanism of mitochondrial Ca^2+^ accumulation in plants, as discussed below. The animal MCU core forms a complex with a number of interacting proteins essential for its proper function: the splice variant MCUb, essential MCU regulator (EMRE), MCUR1, MICU1, and MICU2 (Wagner et al., 2016). In principle, MCU is similarly permeable to Ca^2+^ and Mn^2+^, like most other Ca^2+^ channel proteins, but in this case, the enigma, how the homeostasis of both ions can be separated, has been solved. Selectivity for Ca^2+^ is conferred by MICU1, which possesses Ca^2+^-binding EF-hands (Kamer et al., 2018). At low [Ca^2+^]_cyt_, MICU1 inhibits MCU (Perocchi et al., 2010). This inhibition is relieved by Ca^2+^ binding at increased [Ca^2+^]_cyt_. Mn^2+^ is also bound by MICU1 with high affinity, but is unable to exert the Ca^2+^-specific structural changes that activate MCU (Kamer et al., 2018). In consequence, Mn^2+^ can only permeate MCU upon elevated [Ca^2+^]_cyt_, but not upon elevated [Mn^2+^]_cyt_, which safeguards the mitochondria from Mn^2+^ overload. Accordingly, mutation of *MCU* renders cells more resistant, and mutation of *MICU1* more sensitive to Mn^2+^ (Kamer et al., 2018).

Besides facilitated Ca^2+^ influx by MCU, animal mitochondria are able to accumulate Ca^2+^ with high affinity. A protein localized to the mitochondrial inner membrane, leucine zipper EF-hand-containing transmembrane protein 1 (LETM1), has been identified to mediate this uptake of Ca^2+^ in exchange of H^+^ at [Ca^2+^]_cyt_ < 1 µM (Jiang et al., 2009). As described below, its activity is reversible, the direction being determined by the pH and [Ca^2+^] gradients. Albeit LETM1 was initially described as K^+^/H^+^ antiporter, reconstitution in proteoliposomes unequivocally demonstrated an electroneutral Ca^2+^/2H^+^ antiport activity of this protein (Tsai et al., 2014). Aberrances in K^+^ homeostasis in *letm1* mutant mitochondria may be explained by the stimulation of Ca^2+^-activated K^+^ channels at higher [Ca^2+^]_mito_ (Jiang et al., 2009). Competition assays further demonstrated that Mn^2+^ is also transported with micromolar affinity, pointing to an additional role of LETM1 in Mn^2+^ homeostasis and Ca^2+^/Mn^2+^ interaction.

Largely based on findings in animals, Ca^2+^ transport mechanisms in plant mitochondria are being unveiled (Carraretto et al., 2016; Wagner et al., 2016). Based on genome information, the MCU complex can serve as blueprint for a mitochondrial Ca^2+^ import mechanism in plants, albeit some of its proteins, like EMRE, are not present in plant genomes. In contrast, MCU itself has fanned out: the Arabidopsis genome harbors six genes encoding MCU homologs. As described above, at least one member of the family, cMCU, is localized in the chloroplast as well as in root mitochondria. A member with mitochondrial localization, MCU1, shows Ca^2+^ channel activity upon reconstitution in planar lipid bilayers (Teardo et al., 2017). It is inhibited by Gadolinium and Ruthenium Red, which resembles its animal counterpart, and it is downregulated by MICU. Knockout mutants for this gene have a reduced root length and altered mitochondrial morphology, but only slight changes in mitochondrial Ca^2+^ dynamics, which implies the activity of redundant Ca^2+^ influx pathways. This needs to be solved by multiple knockout mutants. For Arabidopsis MCU1 and MCU2, mitochondrial Ca^2+^ influx has also been shown by yeast complementation (Selles et al., 2018), and MCU2 has been functionally expressed in HEK293 cells (Tsai et al., 2016). Both proteins are present in pollen mitochondria, and pollen germination of *mcu2* is impaired, indicating the importance of [Ca^2+^]_mito_ in this process, which requires further clarification.

The only homolog of MICU in Arabidopsis binds Ca^2+^ through three EF-hands, like its animal counterparts (Wagner et al., 2015). Responses of [Ca^2+^]_mito_ to ATP and auxin were elevated in *micu* knockout mutants, which is explained by the function of Arabidopsis MICU as a negative regulator of mitochondrial Ca^2+^ influx (Wagner et al., 2015). Its knockout alters mitochondrial ultrastructure and causes changes in the respiratory machinery, that, however, do not provoke defects in whole-plant development. It is tempting to speculate that one role of MICU in plants lies in the discrimination of Ca^2+^ and Mn^2+^, like that of its mammalian counterpart. This also provokes the question to which extent plant MCU complexes contribute to mitochondrial Mn^2+^ uptake.

Apart from MCU complexes, a member of the GLR family, GLR3.5, has been localized to the IMM in Arabidopsis (Teardo et al., 2015). This channel is dually targeted, whereby two splice variants encode proteins that are directed either to chloroplasts or to mitochondria. Mitochondrial ultrastructure is altered and mitochondrial Ca^2+^ uptake is slightly reduced in a knockout mutant. It remains to be demonstrated whether GLR3.5 functions as a Ca^2+^ channel or whether it indirectly regulates Ca^2+^ fluxes mediated by other channels. In addition, GLR3.5 has also been shown to localize to the plasma membrane and to co-operate with GLR3.1 in guard cells (Kong et al., 2016).

A potential pathway for sequential Mn^2+^ uptake into the matrix of animal mitochondria consists of two poorly selective Fe^2+^ transporters, divalent metal transporter 1 (DMT1; synon. NRAMP2) and mitoferrin 1 (Mfrn1). There is evidence that DMT1 transports Mn^2+^ from the cytosol across the OMM to the intermembrane space (Wolff et al., 2018). This is intriguing because DMT1 has been described before as an endosomal transporter that translocates metals *into* the cytosol, and because the OMM is considered to be unselectively permeable to solutes. A homolog of DMT1 in Arabidopsis, NRAMP2, functions as Mn^2+^ transporter in the TGN/EE compartment, as described above. There is no evidence of its localization in mitochondria, and mitochondrial MnSOD is not altered in *nramp2* mutants (Alejandro et al., 2017). Mn^2+^ flux across the IMM into the mitochondrial matrix of animals is mediated by Mfrn1, which belongs to the mitochondrial carrier family (Christenson et al., 2018). Interestingly, in Arabidopsis, two homologs to Mfrn1, mitochondrial iron transporter1 (MIT1) and MIT2, were recently described as essential mitochondrial Fe^2+^ transporters (Jain et al., 2019). These are very promising candidates for a metal-selective high-affinity Mn^2+^ influx pathway alongside a low-affinity influx by MCUs.

### Release of Ca^2+^ and Mn^2+^

In mammals, Ca^2+^ release mechanisms counteract the voltage-driven passive accumulation of Ca^2+^ in the mitochondrial matrix mediated by MCU. In excitable cells, matrix Ca^2+^ is released by the Na^+^/Ca^2+^ exchanger NCLX (Luongo et al., 2017). Knockout mice for this gene displayed mitochondrial Ca^2+^ overload causing superoxide generation, necrosis and, eventually, cardiac failure, which demonstrates that mitochondrial Ca^2+^ efflux is essential for mitochondrial function and cell survival.

Another mechanism to release Ca^2+^ from the mitochondrial matrix is based on LETM1, which mediates bidirectional mitochondrial Ca^2+^/2H^+^ antiport, as described above (Jiang et al., 2009). An absence of LETM1 therefore not only results in reduced Ca^2+^ uptake by mitochondria at low [Ca^2+^]_cyt_, but also in a Ca^2+^ overload of mitochondria that accumulate Ca^2+^ by MCU activity. As LETM1 is essential to maintain [Ca^2+^]_mito_ homeostasis, knockout of LETM1 is embryo-lethal in mice and causes severe metabolic and neurological defects in humans (Jiang et al., 2013). Owing to its affinity for Mn^2+^, LETM1 may also release this cation when operating in reverse direction, what remains to be studied.

Two homologs of LETM1 have been described in Arabidopsis (Zhang et al., 2012a). The double knockout mutation of these genes is lethal; developing seeds are aborted. In hemizygous plants, the abundance of mitochondrial proteins is affected and growth is reduced. These phenotypes suggest a conservation of LETM1 function in the homeostasis of [Ca^2+^]_mito_ and possibly [Mn^2+^]_mito_ of plants, what remains to be investigated.

## Ca^2+^ and Mn^2+^ in the peroxisome

### Functions of Ca^2+^

Peroxisomes play numerous roles in metabolism and development, which have been the subject of a recent review (Pan et al., 2020). Ca^2+^ appears to function in intra-peroxisomal signaling, since they contain Ca^2+^ sensor proteins which regulate diverse metabolic processes. These include Ca^2+^-dependent protein kinases, such as GhCPK33, which regulates JA biosynthesis in cotton (Hu et al., 2018), and PiCDPK2, that regulates pollen tube growth in petunia (Guo et al., 2013), as well as the CaM-like protein CML3, which mediates dimerization of the DEG15 protease in Arabidopsis (Dolze et al., 2013). A peroxisomal catalase, Cat3, which degrades H_2_O_2_ into H_2_O and O_2_, is also activated by Ca^2+^/CaM (Yang and Poovaiah, 2002). The identity of the regulatory CaM is unknown, with CML3 being a candidate. This relationship is supported by an increase of guard cell peroxisomal H_2_O_2_ scavenging activity upon increased [Ca^2+^]_peroxi_ (Costa et al., 2010). The peroxisomal import and activity of arginine-dependent NO synthase (NOS) also require Ca^2+^/CaM (Corpas and Barroso, 2014). However, the identity of the NOS, as well as the mechanisms of its regulation by Ca^2+^/CaM, is unknown.

### Functions of Mn^2+^

Peroxisomes are important in the detoxification of free radicals (Pan et al., 2020). For some plant species, such as pea, watermelon, cucumber, castor bean, and carnation, a MnSOD has been shown to be present in plant peroxisomes (Corpas et al., 2017).

### Import and release of Ca^2+^ and Mn^2+^

The presence of Ca^2+^ signal-decoding machinery, as well as a Mn^2+^-dependent SOD, implies the operation of transport mechanisms for those cations in the peroxisomal membrane. This is supported by peroxisomal [Ca^2+^] measurements showing that [Ca^2+^]_peroxi_ dynamics follow those of [Ca^2+^]_cyt_ upon stimulation (Costa et al., 2010).

No membrane proteins that mediate those fluxes are known to date. However, plant peroxisomes contain a protein of the MVP17/PMP22 family, members of which from mammals and yeast form a poorly selective channel with a wide pore, which is permeable to ions and metabolites (see Charton et al., 2019 for review). Albeit channel activity of a plant PMP22 has yet to be demonstrated, this protein is a likely basis for Ca^2+^ and Mn^2+^ fluxes across the peroxisomal membrane.

## Conclusions and outlook

Ca^2+^ and Mn^2+^ govern a plethora of processes in organellar compartments of plants, the most prominent of which were covered in this review. Ca^2+^ acts as signaling agent within organelles, like in the cytosol, and Mn^2+^ may interfere with this function owing to its ability to (unproductively?) occupy Ca^2+^-binding domains. Both cations also cooperate, as in the OEC. Such functions and interactions of Ca^2+^ and Mn^2+^ are determined by their free concentrations in the different compartments, which might be examined by genetically encoded indicators, that need yet to be developed for Mn^2+^. This issue may also be approached by direct subcellular imaging of Ca^2+^ and Mn^2+^, for example, by synchrotron radiation, but such techniques with subcellular resolution are not widely available, extraordinarily complex, and unable to show dynamic changes. Both approaches need to be pushed forward (see Advances and Outstanding Questions).

Membrane transport proteins are the backbone of cellular cation (re-)distribution, and in the last 5 years, a number of new players have shown up on the pitch. Notably, routes for Ca^2+^ and Mn^2+^ into chloroplasts and mitochondria are now known, albeit not fully understood, and further candidates are lining up to be tested. The plant ER/NE has turned out as a hub of Ca^2+^ signaling in different circumstances, and channel proteins in this compartment have been found: The identification of CNGCs in the NE solved the long-standing question how nuclear Ca^2+^ signals are generated in symbiotic signaling, which has very recently taken another turn with the characterization of CASTOR as Ca^2+^ channel. The finding that GLRs essential for systemic Ca^2+^ signaling may also reside in ER and vacuole, besides their operation in the plasma membrane, demands a scrutiny of their roles in those endomembranes. In this respect, the function and regulation of the most-studied plant channel, TPC1, is also far from clear. Some major gaps in the array of organellar transporters still need to be filled. For example, in the plant Golgi, no transporters associated with Mn^2+^ import for glycosylation and no Ca^2+^ release channels are known; peroxisomal Ca^2+^ and Mn^2+^ transport is a black box.

Mechanisms on protein, organellar, and cellular levels ought to be in place that define the distribution and ratio of Ca^2+^ and Mn^2+^. Here, tunable discrimination mechanisms have become apparent from nonplant work in the form of EF-hands, either in accessory subunits (as in animal MCU-MICU) or in the transport protein itself (as in yeast PMR1 or human SPCA1). Conversely, for the chloroplastic BICAT proteins that supply the organelle with Mn^2+^ and shape its Ca^2+^ signals, mechanisms of tell Mn^2+^ from Ca^2+^ are unknown, and even the mode of transport of this family is debated. The search for interactors, transport assays on proteoliposomes, and eventually the solving of a protein structure will lead toward a better understanding.

Finally, the way Ca^2+^ and Mn^2+^ are allocated to organelles is far from clear. How do organelles get prioritized? Are there hard-wired logics? To test the simplest assumption that substrate allocation is determined by different affinities of the transport proteins, their kinetics need to be determined, which is not trivial. However, a more sophisticated coordination and communication between compartments and subcompartments seem inevitable for an efficient utilization of the cations and the optimum supply of target proteins. An impressive example of coordinated ion concentrations is the animal secretory pathway with its subcompartmental [Ca^2+^] gradient. Yet there is some evidence for specific supply chains in plants. Mn^2+^ delivery to chloroplasts, for example, is believed to require sequential Mn^2+^ release from the TGN and the vacuole by NRAMP2 and NRAMP3/4, respectively. This bears some similarity to yeast, where the NRAMP2 homolog SMF2 supplies the mitochondria by endosomal Mn^2+^ release.

What guarantees that these intraorganellar fluxes reach the correct recipient? Besides spatial proximity, membrane contact sites, at which Ca^2+^ or Mn^2+^ are passed from one organelle to the next, are likely to play a major role that has yet to be unveiled in plants. Examples from yeast and animals are in support of this, for instance between ER and mitochondria, where Ca^2+^ transport proteins of different organelles even physically interact.

After completing the toolbox of Ca^2+^ and Mn^2+^ transport, the regulation of those proteins and their interaction within and between organelles needs to be resolved to reach an understanding of Ca^2+^ and Mn^2+^ handling by the plant cell. Sneaking a peek at animals and yeast has proven fruitful in the past and will yield more insights in the future.

**ADVANCES**

- The molecular basis of many Ca^2+^ and Mn^2+^ transport activities across organellar membranes has been revealed. In particular, transporters for Ca^2+^ and Mn^2+^, and a Ca^2+^ channel, have recently been identified in the chloroplast.
- The contribution of organelles, in particular the ER and the vacuole, to cytosolic Ca^2+^ signaling in plants has become evident.
- Transporters that detoxify Mn^2+^ have been identified in the Golgi and the vacuole.
- A role of the TGN in cellular Mn^2+^ distribution has become apparent.

**OUTSTANDING QUESTIONS**

- What is the identity of the missing transport proteins, e.g. to release Ca^2+^ from the Golgi, or to move Ca^2+^ and Mn^2+^ across peroxisomal membranes? Which transport proteins supply the Golgi and ER with Ca^2+^ and Mn^2+^ for glycosylation processes?
- What are the (sub)organellar concentrations of total and free Ca^2+^ and Mn^2+^? Is there a gradient of Ca^2+^ along the secretory pathway, as there is in animals?
- How are Mn^2+^ and Ca^2+^ homeostasis controlled individually if transport proteins are unspecific?
- What is the contribution of different organelles to specific Ca^2+^ signals?
- How is the vacuolar cation channel TPC1 integrated in systemic Ca^2+^ signaling?
- Does Mn^2+^ interfere with Ca^2+^-dependent signaling processes, and does it have a signaling function on its own?
- How are Ca^2+^ and Mn^2+^ allocated to different organelles, especially under limiting conditions?

## Notes

E.P. conceived the article. J.H., N.R., M.T.T.H., S.A., and E.P. wrote the article. E.P. agrees to serve as the author responsible for contact and ensures communication.

The author responsible for distribution of materials integral to the findings presented in this article in accordance with the policy described in the Instructions for Author (https://academic.oup.com/plphys/pages/general-instructions) is: Edgar Peiter (edgar.peiter@landw.uni-halle.de).
